# Meta‐analysis and transcriptomic analysis reveal that NKRF and ZBTB17 regulate the NF‐κB signaling pathway, contributing to the shared molecular mechanisms of Alzheimer's disease and atherosclerosis

**DOI:** 10.1111/cns.14683

**Published:** 2024-05-13

**Authors:** Di Zhang, Keyan Chen, Li Shen Shan

**Affiliations:** ^1^ Department of Cardiology Shengjing Hospital of China Medical University Shenyang Liaoning China; ^2^ Laboratory Animal Science of China Medical University Shenyang Liaoning China; ^3^ Department of Pediatrics Shengjing Hospital of China Medical University Shenyang Liaoning China

**Keywords:** Alzheimer's disease, atherosclerosis, meta‐analysis, NKRF, ZBTB17

## Abstract

**Introduction:**

Alzheimer's disease (AD) and atherosclerosis (AS) are widespread diseases predominantly observed in the elderly population. Despite their prevalence, the underlying molecular interconnections between these two conditions are not well understood.

**Methods:**

Utilizing meta‐analysis, bioinformatics methodologies, and the GEO database, we systematically analyzed transcriptome data to pinpoint key genes concurrently differentially expressed in AD and AS. Our experimental validations in mouse models highlighted the prominence of two genes, NKRF (NF‐κB‐repressing factor) and ZBTB17 (MYC‐interacting zinc‐finger protein 1).

**Results:**

These genes appear to influence the progression of both AD and AS by modulating the NF‐κB signaling pathway, as confirmed through subsequent in vitro and in vivo studies.

**Conclusions:**

This research uncovers a novel shared molecular pathway between AD and AS, underscoring the significant roles of NKRF and ZBTB17 in the pathogenesis of these disorders.

## Graphical abstract

NKRF and ZBTB17 participate in the progression of AD and AS by regulating the NF‐κB signaling pathway.

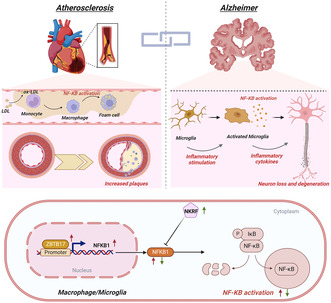

## 1 INTRODUCTION

Alzheimer's disease (AD) and atherosclerosis (AS) are major health challenges, especially in the elderly, with significant socio‐economic impacts.^1^ AD is a progressive neurodegenerative disorder causing cognitive and memory decline, while AS is characterized by chronic vascular inflammation and lipid accumulation, leading to vascular issues.^2^, ^3^ Despite extensive research, the molecular links between AD and AS are not fully understood, hindering the development of effective treatments.

Recent advances in big data, particularly transcriptome analysis, have opened new avenues for exploring disease etiologies. This approach involves examining gene expression in disease states, helping identify disease‐linked genes and pathways through meta‐analysis of multiple data sets.^4^, ^5^, ^6^ A key focus is the NF‐κB signaling pathway, known to be involved in both AD and AS, and its interaction with differentially expressed genes in these diseases.^7^, ^8^

Mouse models, especially with CRISPR technology, are instrumental in studying disease progression and gene‐function relationships. These models, combined with high‐throughput sequencing, provide detailed insights into the transcriptomic changes following gene modifications.^9^, ^10^, ^11^

Our study aims to integrate bioinformatics, transcriptome analysis, and mouse model research to explore the molecular connections between AD and AS. We focus on the NF‐κB pathway and differentially expressed genes to understand shared mechanisms and potentially identify new therapeutic targets for both diseases.

## 2 MATERIALS AND METHODS

### 2.1 Data acquisition

AD and AS‐related transcriptome datasets were retrieved from the GEO database. Details of the datasets are in Table S1.

### 2.2 Differential gene expression analysis

Differential gene expression in AD (datasets GSE63060, GSE138260) and AS (GSE100927) was analyzed using R's “limma” package.^12^ A significance level of *p* < 0.05 was set for filtering. Heatmaps and volcano plots were generated using R's “heatmap” and “ggplot2” packages, respectively.

### 2.3 Meta‐analysis

Meta‐analysis was performed on eight datasets using R's “meta” package. The standard mean difference (SMD) and 95% confidence interval (95% CI) were used for analysis, with a fixed‐effect model applied if *p* > 0.05 and *I* ^2^ < 50%, and a random‐effects model for *p* < 0.05 and *I* ^2^ > 50%. Heterogeneity was assessed using *Q* test and *I* ^2^.^13^

### 2.4 ROC curve analysis

ROC curves were plotted using R's “pROC” package to assess the accuracy of disease status determination based on gene expression.^14^

### 2.5 Mouse model construction and sample collection

Mouse models used were 3×Tg (AD model), ApoE^−/−^, and genetically modified NKRF and ZBTB17 mice, created using CRISPR. Only 6‐week‐old male mice were used. Genotyping was done via PCR of tail DNA. Mice were housed under controlled conditions and the AS model mice were given a high‐fat diet. Sample collection involved brain and aorta tissues, blood, serum, and PBMCs, with various established methods.^15^

Groups for gene expression validation and disease progression studies included WT, 3×Tg, ApoE^−/−^ + HFD, and various combinations with NKRF and ZBTB17 modifications, with 10 mice per group. Randomization and blinding techniques were employed in the animal experiments. This study has been approved by the Laboratory Animal Welfare and Ethic Committee of China Medical University (CMUXN2023061).

### 2.6 Morris Water Maze

Mice underwent a Morris Water Maze test, including a 2‐day visible platform phase, a 5‐day hidden platform phase, and a day for platform exploration.^16^ Motor abilities and exploration behavior of mice were evaluated using open field and object recognition tests. Trials were conducted three times a day, with a maximum duration of 60 s. Mice failing to reach the platform were guided manually. The VisuTrack system recorded data such as incubation period and swimming speed.

### 2.7 Culturing and transfecting microglia and macrophages

HMC3 and THP‐1 cell lines were cultured and differentiated into macrophages. NKRF knockdown and ZBTB17 overexpression were achieved using lentiviral transduction.^17^ Lentivirus‐packaged HMC3 and THP‐1 cells were transfected at 70‐90% confluency and selected for resistance with puromycin (Table S2).

### 2.8 In vitro phagocytosis assay

HMC3 cells were treated with fluorescently labeled *E. coli* and analyzed for phagocytosis using a fluorescence spectrophotometer.^18^

### 2.9 Immunohistochemical staining

Mouse brain and aortic tissues were stained to detect microglia, macrophages, β‐amyloid. Semi‐quantitative analysis was conducted using Image pro plus software.^19^ Immunofluorescence staining was used for detecting co‐expression of NKRF and ZBTB17 with glial and macrophage markers.

### 2.10 TUNEL staining

Neuronal apoptosis in mouse hippocampal tissue was detected using the Click‐iT Plus TUNEL Assay Kit. NeuN labeling identified neurons, with observation methods similar to immunofluorescent staining.^20^

### 2.11 ELISA

Levels of Aβ40, Aβ42, IL‐1β, and TNF‐α in cell culture supernatants, mouse serum, and homogenized mouse brain tissue samples were measured using ELISA kits from Thermo Fisher and Abcam.^21^

### 2.12 Oil red O staining

Post‐euthanasia, mouse aortas were collected, stained with oil red O, and observed using an Olympus CX43 microscope. Semi‐quantitative analysis was done with Image pro plus software.^15^

### 2.13 Blood lipid and oxidative stress level detection

Serum triglyceride (TG) levels and malondialdehyde (MDA) levels in mouse brain homogenate and serum were measured using kits from Solarbio.^22^

### 2.14 RT‐qPCR

Total RNA was extracted and converted to cDNA. RT‐qPCR was conducted using the ABI 7500 system, with GAPDH as the internal reference. The 2^−ΔΔCt^ method quantified gene expression changes (Table S3).^23^

### 2.15 Western blot

Protein was extracted and quantified from samples. SDS‐PAGE and electrophoresis were followed by transfer to a PVDF membrane. After incubation with primary and secondary antibodies, the membrane was developed using ECL solution and analyzed. The experiment was repeated thrice (Table S4).^24^

### 2.16 Dual‐luciferase assay

HMC3 cells were transfected with oe‐NC, oe‐ZBTB17, and NFKB1 promoter reporter vectors (wild‐type and mutants) to assess ZBTB17's effect on NFKB1 transcription. Luciferase activity was measured 48 h post‐transfection using BioVision's Luciferase Assay Kit. The Promega system calculated the ratio of firefly to Renilla luciferase for gene activation levels.^25^

### 2.17 ChIP experiment

A ChIP assay was performed to study ZBTB17's enrichment in the NFKB1 gene promoter using Sicheng Biotechnology's kit. Cells were cross‐linked and sonicated, and the DNA‐protein complexes were immunoprecipitated with anti‐ZBTB17. DNA fragments were recovered and analyzed via qPCR to detect NFKB1 promoter regions.^26^, ^27^

### 2.18 Statistical analysis

Bioinformatics data were analyzed using R 4.2.2, while other results were processed with SPSS 26.0. Data are presented as mean ± standard deviation. The Shapiro‐Milk test checked for normality and variance homogeneity. Independent *t*‐tests, one‐way ANOVA, and repeated measures analysis were used based on data distribution and group comparisons, with *p* < 0.05 as the significance threshold.

## 3 RESULTS

### 3.1 21 DEGs consistent in AD and AS

**FIGURE 1:**
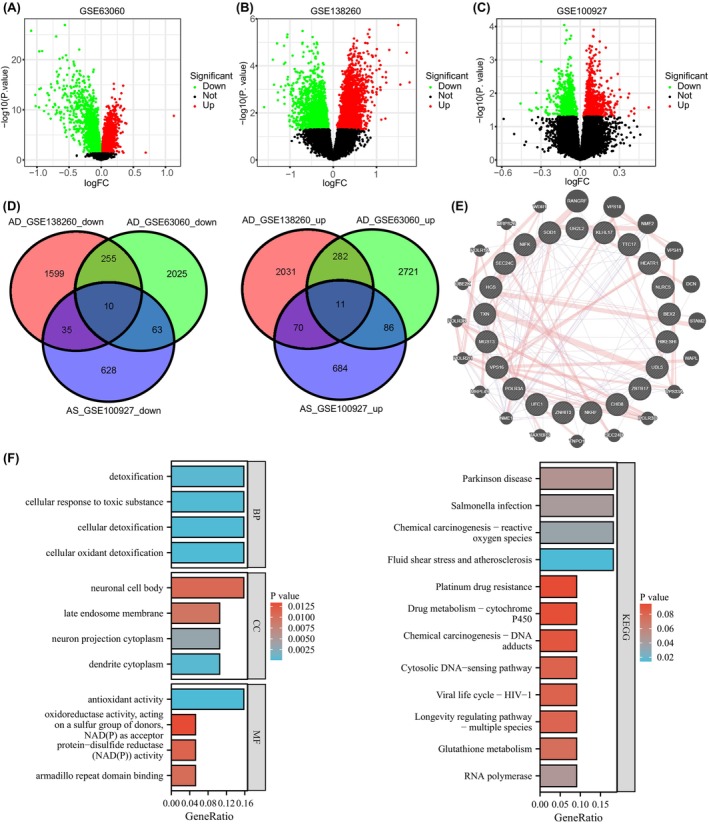
Joint DEGs screening and functional enrichment analysis of AD and AS. (A–C) Volcano plots of differentially expressed genes in datasets GSE63060 (Control = 104, disease = 145), GSE138260 (Control = 19, Disease = 17), and GSE100927 (Control = 35, disease = 69) are shown, with red dots representing upregulated genes and green dots representing downregulated genes. (D) The intersection results of upregulated genes (left) and downregulated genes (right) in datasets GSE63060, GSE138260, and GSE100927 are depicted. An interaction network of the 21 intersecting genes obtained from the GENEMANIA website (E) is illustrated, which includes the interacting genes (outer circle) of the 21 genes provided by the website. The GO and KEGG enrichment results of the intersecting genes are also presented (F).

In our study on Alzheimer's disease (AD) and atherosclerosis (AS), both age‐related inflammatory diseases, we analyzed two AD datasets (GSE63060 and GSE138260) and one AS dataset (GSE100927) from the GEO database (Figure 1A–C). We identified 21 differentially expressed genes (DEGs) with consistent trends across these diseases. Among these, 11 genes (HEATR1, SEC24C, HGS, OR2L2, ZBTB17, NLRC5, VPS16, POLR3A, TTC17, KLHL17, and CHD8) were upregulated, and 10 genes (BEX2, ZNHIT3, UFC1, MGST3, SOD1, UBL5, MK167IP (NIFK), TXN, NKRF, and C11orf73 (HIKESHI)) were downregulated in AD or AS (Figure 1D).

We also constructed an interactome network for these genes using GENEMANIA (Figure 1E) and conducted functional enrichment analysis. The analysis indicated that these genes are mainly involved in processes related to cellular detoxification and response to toxic substances, and are associated with pathways like fluid shear stress in atherosclerosis and chemical carcinogenesis (Figure 1F). This suggests the shared molecular underpinnings in the progression of both AD and AS through these 21 DEGs.

### 3.2 Six key genes involved in the progression of AD and AS were identified through meta‐analysis screening

To further screen genes closely related to Alzheimer's disease (AD) and atherosclerosis (AS), we increased the number of datasets from the GEO database. We performed a meta‐analysis to analyze the expression patterns of the 21 overlapping genes in AD and AS. The analysis results show that out of the 18 genes, which were expressed in eight AD or AS datasets (Figure S1), the 95% confidence interval of 11 genes did not include “0”, indicating significant differences in expression trends (Table S5). KLHL17, NLRC5, SEC24C, and ZBTB17 are upregulated in the disease group (AS or AD), while BEX2, C11orf73 (HIKESHI), NKRF, SOD1, UBL5, UFC1, and ZNHIT3 (zinc finger HIT‐type containing 3) are downregulated in the disease group (Table S5, Figure S2).

**FIGURE 2:**
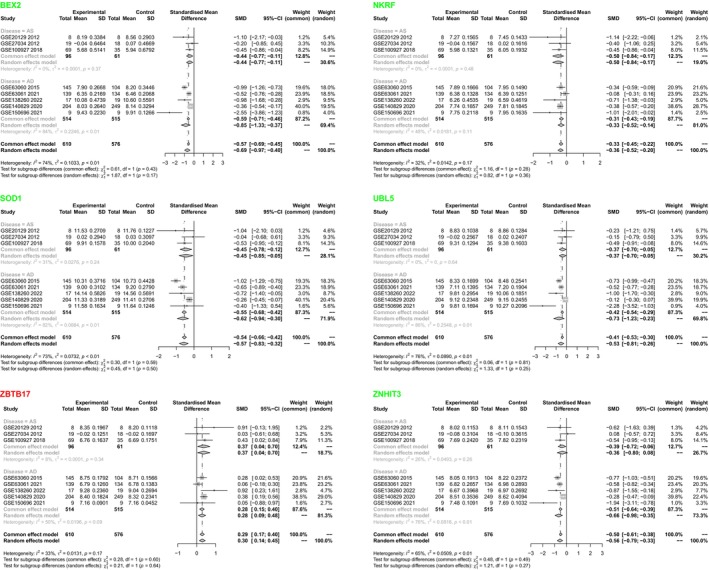
Meta‐analysis results of six genes, including BEX2 in the meta subgroup. The meta subgroup analysis results of BEX2, NKRF, SOD1, UBL5, ZBTB17, and ZNHIT3 are presented here, with disease type as the grouping criterion. Red marks indicate upregulated genes, while green marks indicate downregulated genes.

Subsequently, we performed subgroup analysis on 11 differential genes, using disease (AD and AS) as the grouping criterion. The results showed that six genes had significant differential expression between the AS and AD groups, and their trends were consistent. Among them, NKRF and ZBTB17 had low inter‐group data heterogeneity (the *I* ^2^ values of the AD and AS groups were close and both <50%, compared to AD and AS groups), while BEX2, SOD1, UBL5, and ZNHIT3 had high inter‐group data heterogeneity (Figure 2). Furthermore, we performed a sensitivity analysis to assess the contribution of data heterogeneity sources. The results showed that excluding each dataset did not significantly change *I* ^2^ in the analysis of individual genes (Figure S3A). Therefore, we consider the meta‐analysis results of six genes, including BEX2, relatively reliable.

The ROC curve reflects the discriminatory ability of gene expression between the healthy group and the diseased group. Our analysis results show that in the ROC curves of various datasets, most of the area under the curve (AUC) for six genes, such as BEX2, is greater than 0.5. This result indicates that the expression of these six genes, including BEX2, can distinguish AD and AS (relative to the healthy group), suggesting their involvement in disease progression (Figure S3B).

The above analysis results indicate that BEX2, NKRF, SOD1, UBL5, ZBTB17, and ZNHIT3 exhibit differential expression in both AD and AS, suggesting that they may be key genes involved in the progression of these diseases.

### 3.3 BEX2 and six other genes show differential expression in the pathological tissues of AD and AS mice

**FIGURE 3:**
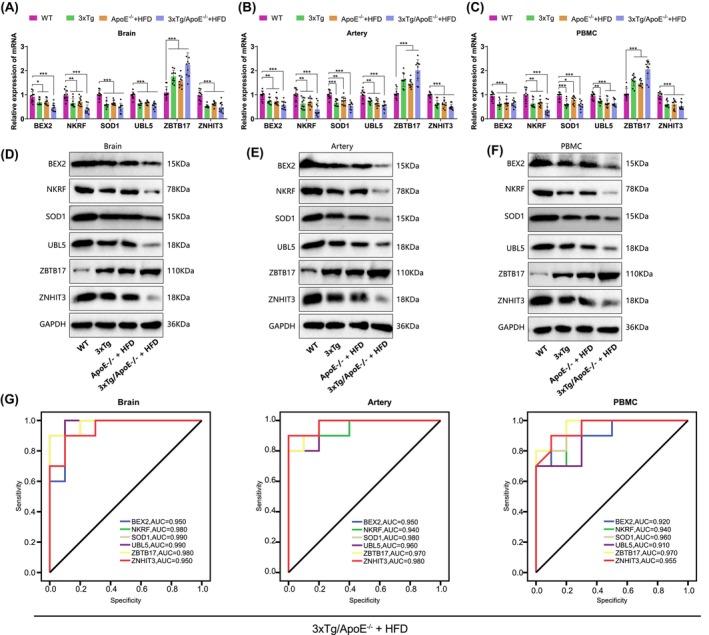
Expression profiles of six genes, including BEX2, in AD and AS mice. (A–F) RT‐qPCR and western blot were used to detect the expression levels of six genes, including BEX2 in brain tissues, aortic tissues, and PBMCs from each group of model mice (*n* = 10 in each group); (G) ROC curves of the RT‐qPCR results of six genes including BEX2 in model mice from the 3×Tg/ApoE^−/−^ + HFD group. ns *p* >0.05, *p < 0.05, **p < 0.01, and ***p < 0.001; The significance values without special markers are obtained by comparing with the control group.

Furthermore, we validated the expression levels of six key genes, including BEX2, in AD and AS through in vitro experiments. As described in the Methods section, we generated AD (3×Tg), AS (ApoE^−/−^ + HFD), and AD combined with AS (3×Tg/ApoE^−/−^ + HFD, AD and AS) mouse models. After 12 weeks of feeding, the mice were euthanized, and samples were collected for analysis. Since the GEO datasets used in the bioinformatics and meta‐analysis part of this study come from different biological samples (mainly brain, artery, and PBMC), we have examined the expression levels of relevant factors in mouse brain tissue, artery tissue, and PBMC. The results of RT‐qPCR and western blot analysis demonstrated that compared to the wild‐type (WT) healthy mice from the same batch of breeding, the levels of BEX2 (*p* < 0.0001, *F* = 12.14), NKRF (*p* < 0.0001, *F* = 15.65), SOD1 (*p* < 0.0001, *F* = 23.85), UBL5 (*p* < 0.0001, *F* = 29.03), and ZNHIT3 (*p* < 0.0001, *F* = 19.45) were significantly downregulated, while ZBTB17 (*P* < 0.0001, F = 20.61) was significantly upregulated in the brain tissue, aortic tissue, and PBMC of the 3×Tg, ApoE^−/−^ + HFD, and 3×Tg/ApoE^−/−^ + HFD groups (Figure 3A–F). These results are consistent with the findings of bioinformatics and meta‐analysis.

Subsequently, we plotted the ROC curve based on the results of RT‐qPCR. The results showed that all genes had a good area under the ROC curve in different tissues (Figure 3G, Figure S4A,B), suggesting that six genes, including BEX2, may be potential biomarkers for diagnosing AD and AS.

The above in vivo validation experiments show that BEX2, NKRF, SOD1, UBL5, ZBTB17, and ZNHIT3 exhibit differential expression in brain tissue, aorta tissue, and PBMC of AD, AS, and AD and AS mice, and their expression patterns are consistent with the results of a meta‐analysis.

### 3.4 NKRF and ZBTB17 are associated with the NF‐κB signaling pathway

We conducted a literature and website review to explore the possible mechanisms of the involvement of six key genes, including BEX2, in AD and AS progression. We discovered that NKRF (NF‐kappa‐B‐repressing factor) is an inhibitor of NF‐κB. It can inhibit the expression of downstream factors of NF‐κB by binding to NF‐κB or interacting with specific negative regulatory elements (5’‐AATTCCTCTGA‐3′).^28^, ^29^ Based on this, we found that among the remaining five genes, ZBTB17 is a transcription factor. The GTRD website (http://gtrd.biouml.org/#!) and JASPAR website (https://jaspar.genereg.net/) predicted that NFKB1 (nuclear factor kappa B subunit 1) is a predicted downstream target of ZBTB17 (Figure S5A,B). ZBTB17 may regulate its expression.

The influence of ZBTB17 on the activity of the NFKB1 promoter was assessed using a dual‐luciferase assay. The results revealed that overexpression of ZBTB17 significantly increased the activity of the wild‐type NFKB1 promoter (NFKB1‐WT) (*p* < 0.0001, *t* = 9.926). However, there was no significant change in the activity of the NFKB1 promoter with a mutated sequence (NFKB1‐MUT) upon overexpression of ZBTB17 (*p* = 0.923, *t* = 0.3679) (Figure S5C). To further investigate whether ZBTB17 binds to the NFKB1 promoter region, we performed chromatin immunoprecipitation experiments (ChIP). The results demonstrated a significant enrichment of ZBTB17 on the NFKB1 promoter upon overexpression (*p* < 0.0001, *t* = 9.575), suggesting that ZBTB17 can bind to the NFKB1 promoter and facilitate its transcription (Figure S5D).

**FIGURE 4:**
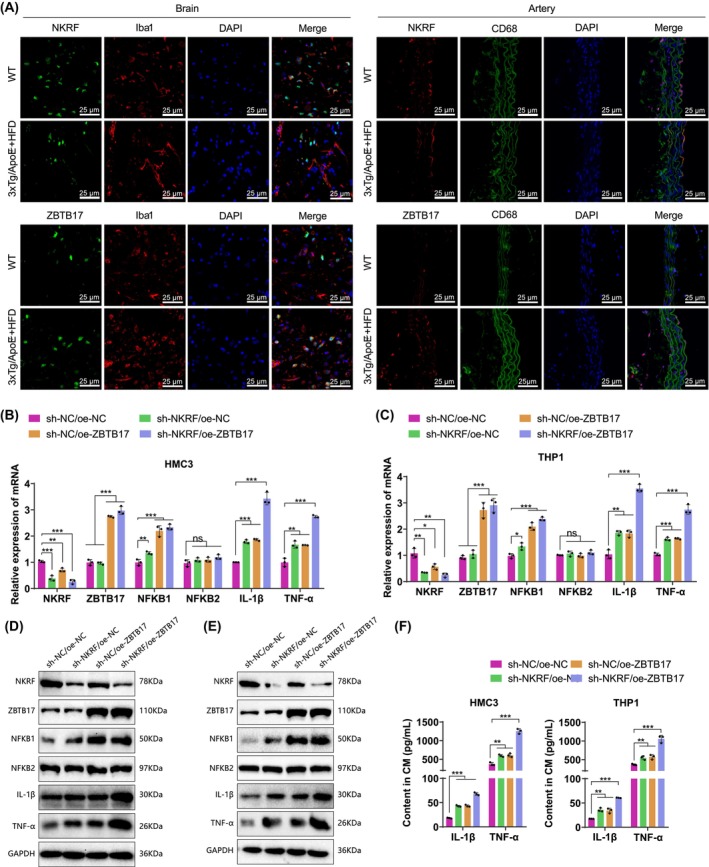
Expression of NKRF, ZBTB17, and related factors in microglia and macrophages. (A) Immunofluorescence staining was used to observe the colocalization of NKRF or ZBTB17 with microglia or macrophages (scale bar = 25 μm); (B–E) RT‐qPCR and western blot were performed to detect the expression of NKRF, ZBTB17, and related factors in HMC3 and THP‐1 cells; (F) ELISA was conducted to measure the expression of IL‐1β and TNF‐α in HMC3 and THP‐1 conditioned media; ns *p* >0.05, *p < 0.05, **p < 0.01, and ***p < 0.001; Significance levels not specifically marked were compared to the control group; Cell experiments were repeated three times.

Previous studies have shown that the activation of NF‐κB and its signaling pathway in astrocytes and macrophages promote the progression of Alzheimer's disease (AD) and atherosclerosis (AS), making them potential targets for the treatment of AD and AS.^30^, ^31^ To verify the expression of NKRF and ZBTB17 in microglia or macrophages of the disease group, we performed immunofluorescence staining on the brain and aortic tissues of gene‐expression‐enriched WT and 3×Tg/ApoE^−/−^ + HFD mice. The results showed that NKRF was expressed at a higher level in the WT group and exhibited more colocalization with microglia or macrophage markers. On the other hand, ZBTB17 was expressed at a higher level in the 3×Tg/ApoE^−/−^ + HFD group and exhibited more colocalization with microglia or macrophage markers (Figure 4A). Furthermore, correlation analysis of six key genes, including BEX2, indicates that NKRF and ZBTB17 exhibit negative expression correlation in multiple datasets (Figure S5C). Therefore, we speculate that NKRF and ZBTB17 may regulate the progression of Alzheimer's disease (AD) and atherosclerosis (AS) by modulating NF‐κB and its related signaling pathways in microglia and macrophages.

To validate the above hypothesis, we performed in vitro experiments using HMC3 (a human microglial cell line) and THP‐1 (a human monocytic cell line induced to differentiate into macrophages) (Figure S5E–G). We separately constructed a knockdown of NKRF (using the sh‐NKRF‐1 sequence) (Figure S5H), overexpressed ZBTB17, and simultaneously overexpressed ZBTB17 in HMC3 and THP‐1 cell lines knocking down NKRF, and then measured the expression levels of NF‐κB and its downstream response factors IL‐1β and TNF‐α in the cell lines. The results of the RT‐qPCR and western blot analyses indicated a significant upregulation in the expression of NFKB1 in the sh‐NKRF/oe‐NC, sh‐NC/oe‐ZBTB17, and sh‐NKRF/oe‐ZBTB17 groups when compared to the sh‐NC/oe‐NC group (*p* < 0.0001, *F* = 73.50). However, there was no significant change observed in the expression of NFKB2 (*p* = 0.127, *F* = 2.57) in comparison to NFKB1. Additionally, the downstream responsive factors of NF‐κB, IL‐1β (*p* < 0.0001, *F* = 319.0), and TNF‐α (*p* < 0.0001, *F* = 185.3) exhibited a significant upregulation (Figure 4B–F).

The above results indicate that NKRF and ZBTB17 can regulate the NF‐κB signaling pathway in microglia and macrophages, which may be a molecular mechanism for their involvement in the progression of AD and AS.

### 3.5 Knocking‐in NKRF or knocking out ZBTB17 can inhibit the progression of AD

Furthermore, we first validated the impact of NKRF and ZBTB17 expression on AD progression, as described in the Methods section. Using CRISPR and hybridization techniques, we generated AD and AD and AS mice with either NKRF knock‐in and/or ZBTB17 knockout. After feeding the mice with HDF for 12 weeks, we collected relevant tissues to assess AD progression.

**FIGURE 5:**
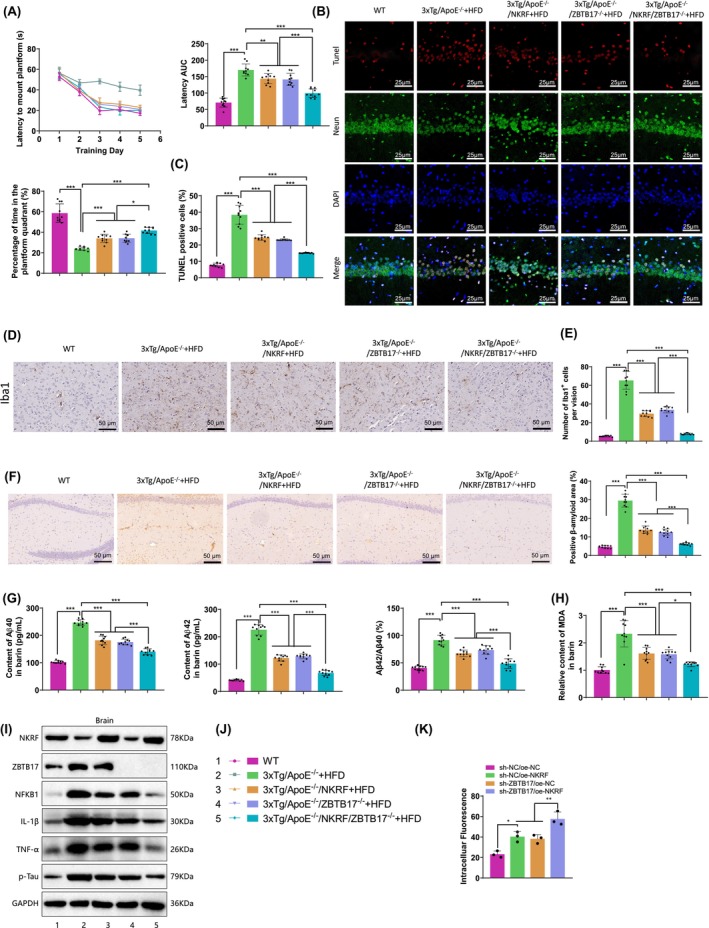
The impact of NKRF and ZBTB17 gene editing on the progression of AD in AD and AS mice. (A) Water maze test was carried out to evaluate the learning and memory capability of mice; (B, C) Immunofluorescence co‐staining was performed to detect the neuronal apoptosis in the hippocampus of mice (scale bar = 25 μm); (D, E) Immunohistochemistry was conducted to determine the number of glial cells in the cortical tissue of mice (scale bar = 50 μm); (F) Immunohistochemistry was used to detect β‐amyloid deposition in the CA1 region of the mouse hippocampus; (G, H) ELISA was used to measure Aβ and MDA levels in the mouse cortex tissue; (I) Western blot analysis was performed to examine the expression of relevant proteins in the cortical tissue of mice; (J) Legend; (K) Testing the phagocytic capability of microglial cells. Each group had *n* = 3; ns *p* >0.05, *p < 0.05, **p < 0.01, and ***p < 0.001; The significance denoted by no special marker indicates comparisons with the control group.

The results of the analysis demonstrated significant inhibition in the progression of Alzheimer's disease (AD) in NKRF knock‐in (NKRF) and ZBTB17 knockout (ZBTB17^−/−^) mice compared to the model mice (3×Tg and 3×Tg/ApoE^−/−^ + HFD). The most notable inhibition of AD progression was observed in mice with simultaneous NKRF knock‐in and ZBTB17 knockout (NKRF/ZBTB17^−/−^) (Figure 5, Figure S6). Specific improvements included enhanced learning and memory capabilities in mice (*p* < 0.0001, *F* = 15.77) (Figure 5A, Figure S6A), reduced neurodegeneration in the hippocampal region (*p* < 0.0001, *F* = 178.4) (Figure 5B,C, Figure S6B,C), decreased population of cortical glial cells (*p* < 0.0001, *F* = 147.4), diminished amyloid deposition in the CA1 region of the hippocampus (*p* < 0.0001, *F* = 272.1), decreased levels of brain cortical Aβ content (Aβ40: *p* < 0.0001, *F* = 238.1; Aβ42: *p* < 0.0001, *F* = 368.3), reduced Aβ42/40 ratio (*p* < 0.0001, *F* = 77.86), lowered oxidative stress levels (as measured by MDA) (*p* < 0.0001, *F* = 38.54), and significantly decreased levels of p‐Tau (S396) (Figure 5D–I, Figure S6D–I). Additionally, western blot analysis revealed that NKRF knock‐in or ZBTB17 knockout resulted in significant downregulation of NFKB1, IL‐1β, and TNF‐α in the cortical tissue of the model mice (Figure 5J, Figure S6J), indicating the involvement of NKRF and ZBTB17 in the regulation of the NF‐κB signaling pathway in the brain tissue (cortex).

To investigate the impact of NKRF and ZBTB17 on the phagocytic function of microglial cells, we examined the phagocytosis of microglial cells overexpressing NKRF or with silenced ZBTB17. We observed a notable increase in the phagocytic ability of microglial cells in the sh‐NC/oe‐NKRF and sh‐ZBTB17/oe‐NC groups compared to the sh‐NC/oe‐NC group. Notably, the sh‐ZBTB17/oe‐NKRF group showed the highest enhancement in phagocytic capacity (*p* < 0.0001, *F* = 24.45) (Figure 5K).

The above results suggest that knocking down NKRF or knocking out ZBTB17 can inhibit the progression of Alzheimer's disease (AD), with the most significant inhibition of AD progression achieved by altering the expression of both factors. This process may be achieved through the regulation of the NF‐κB signaling pathway.

### 3.6 Knocking‐in NKRF or knocking out ZBTB17 can inhibit the progression of AS

Subsequently, we further validated the impact of NKRF and ZBTB17 expression on the progression of AS. Similarly, we generated AS mice with NKRF knock‐in and/or ZBTB17 knockout using CRISPR and hybridization techniques. After breeding the mice for 12 weeks, we collected relevant tissues to assess the progression of AS.

**FIGURE 6:**
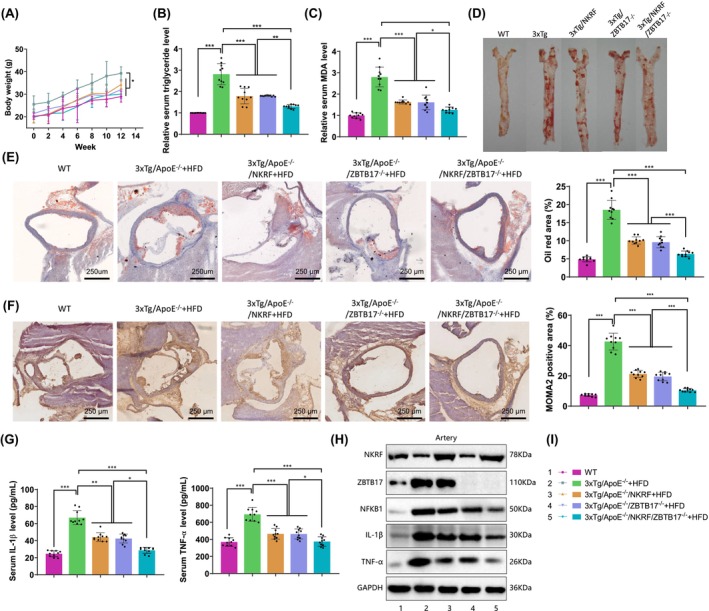
The impact of NKRF and ZBTB17 gene editing on the progression of AS in AD and AS mice. (A) Growth curves of mouse body weight in each treatment group; (B, C) Levels of serum triglycerides and MDA in each group of mice; (D) Representative images of aortic lesions in each group of mice stained with oil red O; (E) Assessment of tissue damage levels in aortic root sections stained with oil red O, with representative images on the left and quantitative statistics on the right (scale bar = 250 μm); (F) Immunohistochemical assessment of macrophage infiltration in aortic tissues, with representative images on the left and quantitative statistics on the right (scale bar = 250 μm); (G) Levels of serum IL‐1β and TNF‐α in each group of mice, as detected by ELISA; (H) Western blot analysis of protein expression in aortic tissues; (I) Legend; *n* = 10 in each group; ns *p* >0.05, *p < 0.05, **p < 0.01, and ***p < 0.001; Significance not otherwise indicated was determined by comparison with the control group.

The test results showed that the progression of AS in NKRF knock‐in (NKRF) and ZBTB17 knockout (ZBTB17^−/−^) mice was significantly inhibited compared to model mice (ApoE^−/−^ + HFD and 3xTg/ApoE^−/−^ + HFD). Moreover, the progression of AS was most prominently inhibited in mice with simultaneous NKRF knock‐in and ZBTB17 knockout (NKRF/ ZBTB17^−/−^) (Figure 5, Figure S6). Specific manifestations included a slowing of weight gain (*p* < 0.0001, *F* = 49.39) (Figure 6A, Figure S7A), a decrease in serum triglycerides (*p* < 0.0001, *F* = 61.57), and a decrease in oxidative stress marker MDA levels (*p* < 0.0001, *F* = 63.95) (Figure 6B,C, Figure S7B,C). There was also a decrease in the number and size of aortic plaques (Figure 6D, Figure S7D), a reduction in aortic root tissue damage area (*p* < 0.0001, *F* = 121.5) (Figure 6E, Figure S7E), a decrease in the number of monocytes‐macrophages in the arteries (*p* < 0.0001, *F* = 124.7) (Figure 6F, Figure S7F), and a decrease in tissue inflammation levels (IL‐1β: *p* < 0.0001, *F* = 95.05; TNF‐α: *p* < 0.0001, *F* = 44.43) (Figure 6G, Figure S7G). Meanwhile, western blot results showed that the expression levels of NFKB1, IL‐1β, and TNF‐α in the aortic tissue of the model mice were significantly reduced after NKRF knock‐in or ZBTB17 knockout (Figure 6H,I, Figure S7H,I), indicating that both NKRF and ZBTB17 are involved in the regulation of the NF‐κB signaling pathway in aortic tissue.

The above results indicate that knocking down NKRF or knocking out ZBTB17 can inhibit the progression of AS. Among them, the inhibition of AS progression is most evident by altering the expression of both genes, and this process may be achieved through the regulation of the NF‐κB signaling.

## 4 DISCUSSION

Our study reveals common molecular pathways in Alzheimer's disease (AD) and atherosclerosis (AS), notably in genes like BEX2, NKRF, SOD1, UBL5, ZBTB17, and ZNHIT3. These genes, identified via meta‐analysis and transcriptome analysis, suggest significant roles in the onset and progression of both AD and AS. Unlike previous research that focused on individual diseases, our approach uncovers potential shared molecular mechanisms^32^, ^33^, ^34^ (Figure S8).

Experimental validation in AD, AS, and AD and AS mouse models confirmed the differential gene expressions found in computational analysis, supporting the robustness of our methods.^35^ Specifically, NKRF and ZBTB17 genes interact with the NF‐κB signaling pathway in microglia and macrophages, key to AD and AS pathogenesis. This finding aligns with known roles of NF‐κB in inflammation and immune responses, suggesting that NKRF and ZBTB17 influence AD and AS progression through NF‐κB pathway modulation in these cells.^8^, ^36^, ^37^, ^38^

Our findings provide a novel and in‐depth understanding of the roles of NKRF and ZBTB17 in AD and AS, setting them apart from previous research. While Ju Hwang et al. (2017) investigated the impact of the NF‐κB signaling pathway in AD,^39^ and Zheng et al. (2022) explored its influence in AS, these studies did not specifically focus on the genes NKRF and ZBTB17.^40^ Importantly, our research explicitly identifies NKRF and ZBTB17 as key molecules that bridge the gap between these two diseases, opening new avenues for understanding the common mechanisms underlying both conditions. Furthermore, while Liu et al. (2020) examined differentially expressed genes in a single AD model,^41^ and Li et al. (2021) conducted a similar study in an AS model,^42^ our study takes a different approach by employing a cross‐disease model. This approach yields new evidence to unravel potential molecular connections between AD and AS, providing crucial insights for the development of therapeutic strategies that target both diseases simultaneously.

Our CRISPR‐based study highlights the therapeutic potential of targeting NKRF and ZBTB17 expressions to manage Alzheimer's disease (AD) and atherosclerosis (AS). Modifying these genes in mouse models showed significant reduction in AD, AS, and combined AD and AS symptoms, likely due to alterations in NF‐κB signaling.^43^ These findings suggest that NKRF and ZBTB17 play key roles in AD and AS progression through their interaction with the NF‐κB pathway in microglia and macrophages. This understanding not only deepens our insight into the molecular mechanisms of AD and AS but also points to potential drug targets, like specific modulators of NKRF and ZBTB17, for treating these conditions.

Yet, as with any scientific inquiry, our study is not without limitations. Relying predominantly on public GEO datasets and laboratory mouse models might not encapsulate the full spectrum and intricacies inherent to human disease contexts. While NKRF and ZBTB17's association with the NF‐κB pathway has been highlighted, the granular molecular interplay remains shrouded, warranting deeper exploration. Future endeavors should aspire to broaden the cohort demographics, encompassing a diverse array of ethnicities, genders, and age brackets, ensuring greater generalizability. Moreover, further exploration is required to determine the exact interaction mechanisms between NKRF and ZBTB17 with the NF‐κB signaling pathway. Specifically, it is necessary to investigate how the expression and functionality of these two genes can be regulated to optimize the activity of the NF‐κB signaling pathway, thus offering new strategies for the treatment of AD and AS.

## AUTHOR CONTRIBUTIONS

DZ, KYC, and LSS wrote the paper and conceived and designed the experiments; DZ and KYC analyzed the data; LSS collected and provided the sample for this study. All authors have read and approved the final submitted manuscript.

## FUNDING INFORMATION

This research was funded by Basic Scientific Research Project of Colleges and Universities of Liaoning Province (Key Program, No. LJKZ0746) and the Basic Scientific Research Project of Colleges and Universities of Liaoning Province (General Program, No. LJKZ0746) and Liaoning Provincial Natural Fund (2022‐MS‐180).

## CONFLICT OF INTEREST STATEMENT

The authors declare no competing interests.

## Supporting information


Data S1.

Figure S1.

Figure S2.

Figure S3.

Figure S4.

Figure S5.

Figure S6.

Figure S7.

Figure S8.

Table S1.

## Data Availability

The data that supports the findings of this study are available on request from the corresponding author.
