# Supplementary material for: Meta‐analysis and transcriptomic analysis reveal that NKRF and ZBTB17 regulate the NF‐κB signaling pathway, contributing to the shared molecular mechanisms of Alzheimer's disease and atherosclerosis
Source: CNS Neurosci Ther. 2024 May 13;30(5):e14683. doi: 10.1111/cns.14683 (PMC11090078; doi:10.1111/cns.14683)
Supplement: Supplementary file 1 — Data S1. [file CNS-30-e14683-s005.docx]

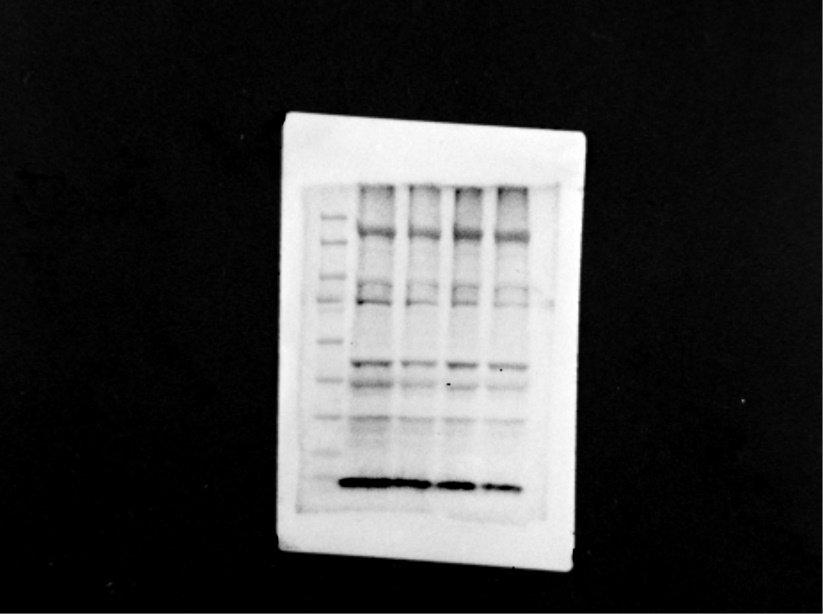


Figure 3D-1


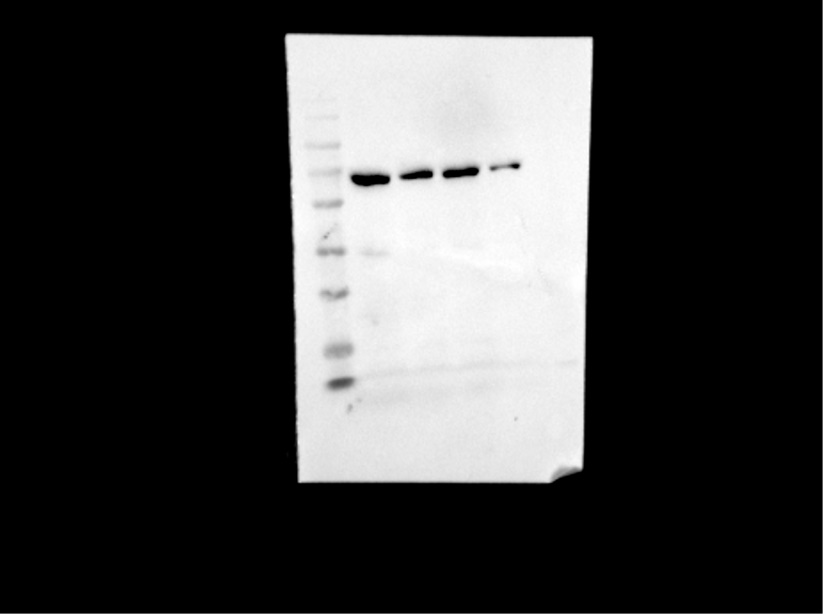


Figure 3D-2


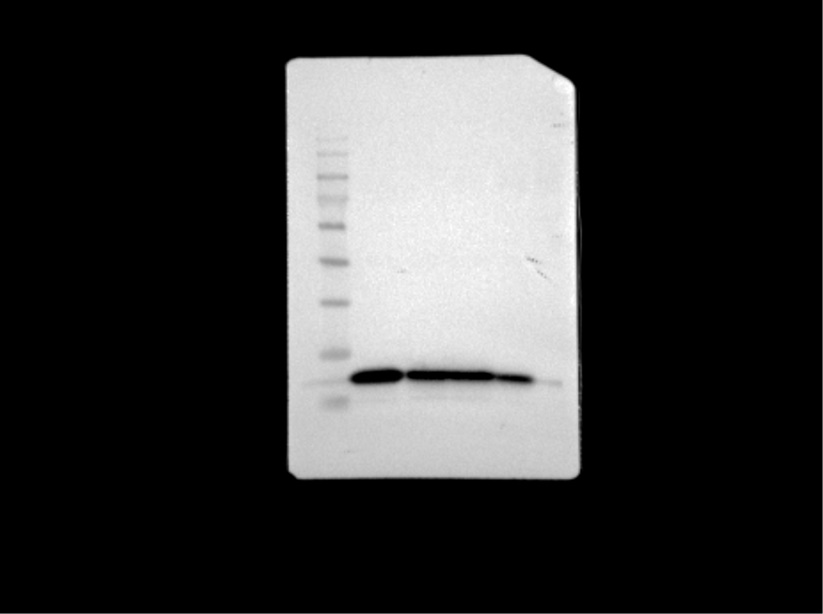


Figure 3D-3


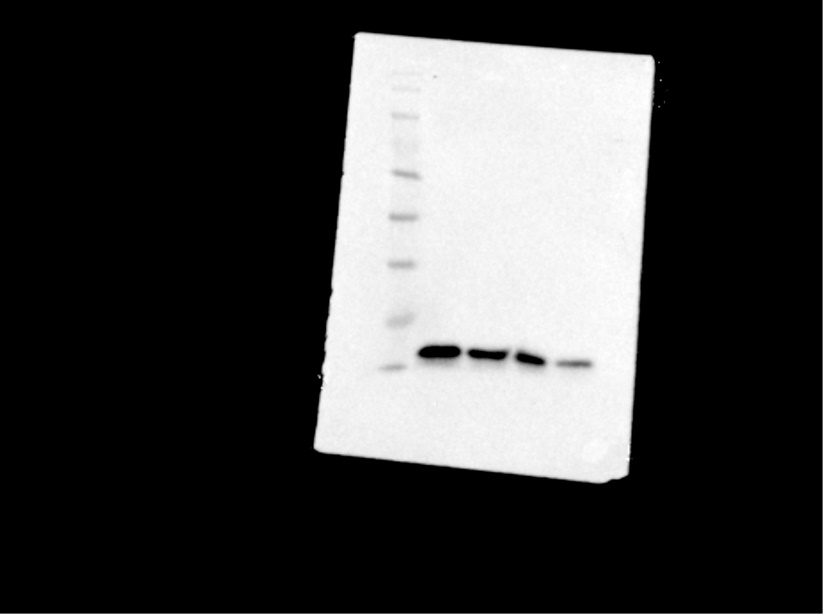


Figure 3D-4


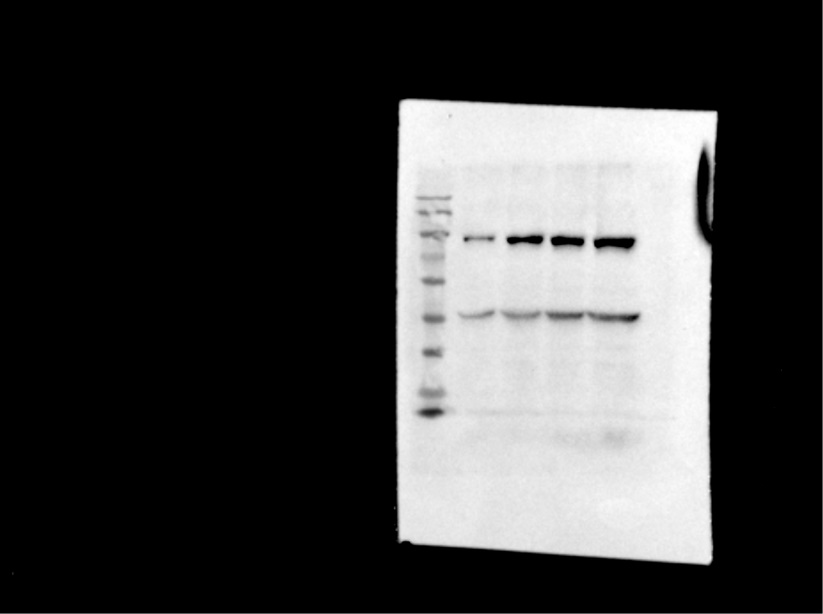


Figure 3D-5


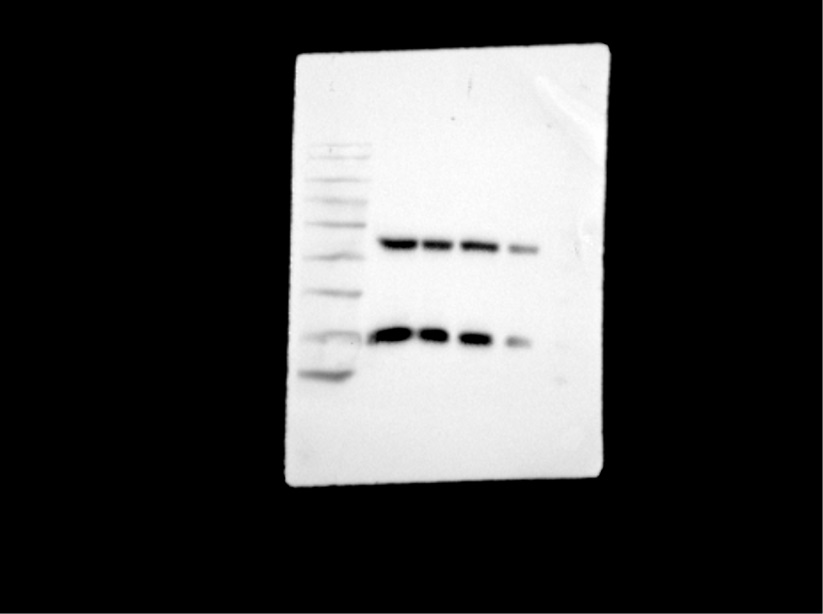


Figure 3D-6


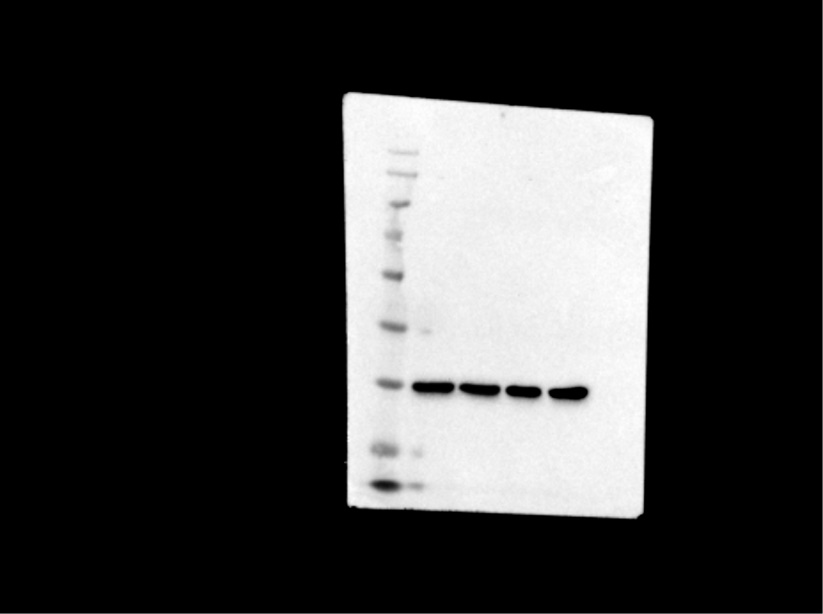


Figure 3D-7


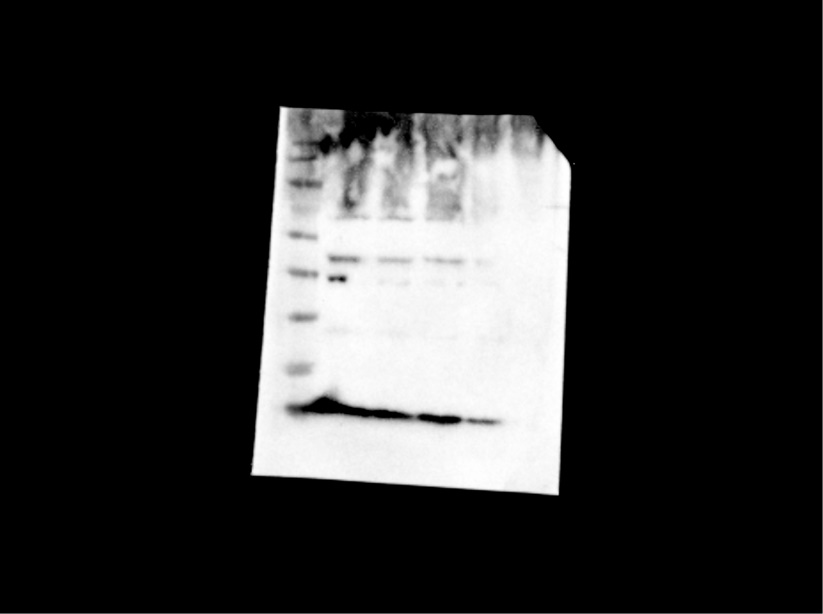


Figure 3E-1


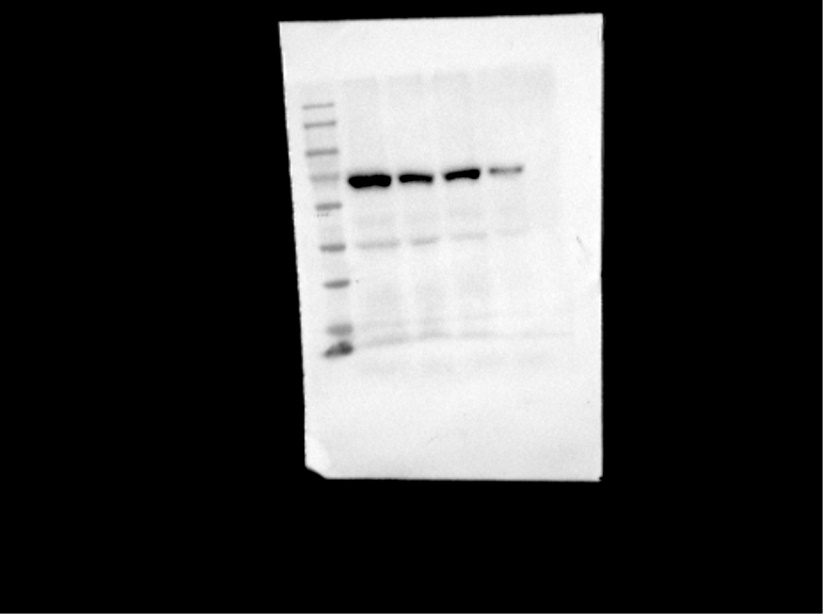


Figure 3E-2


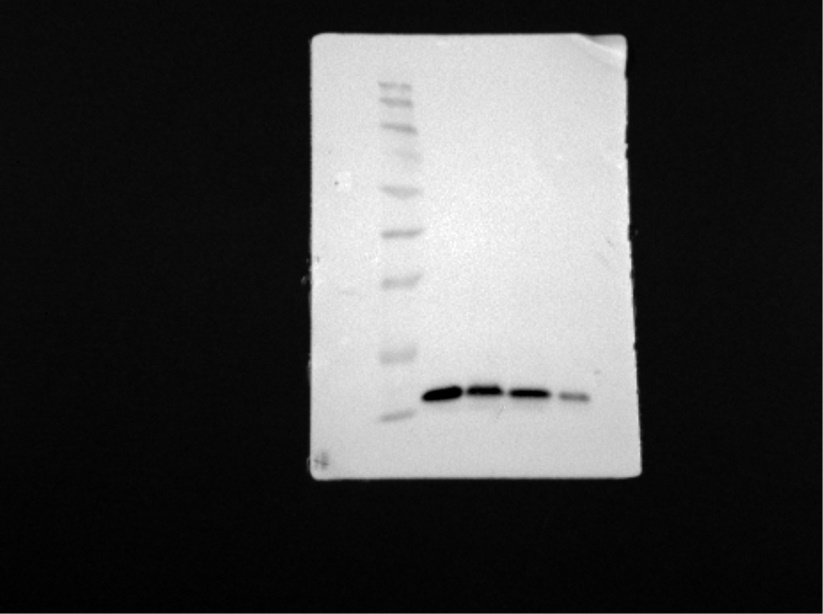


Figure 3E-3


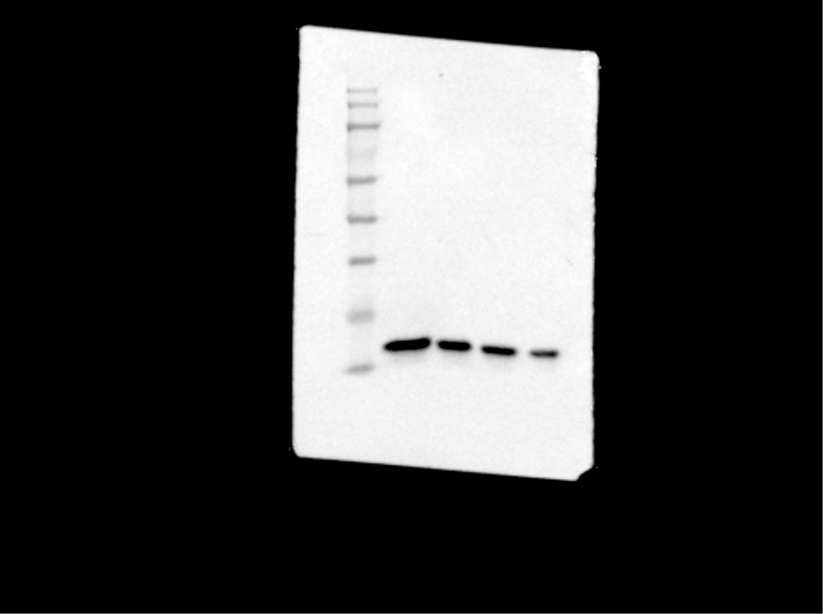


Figure 3E-4


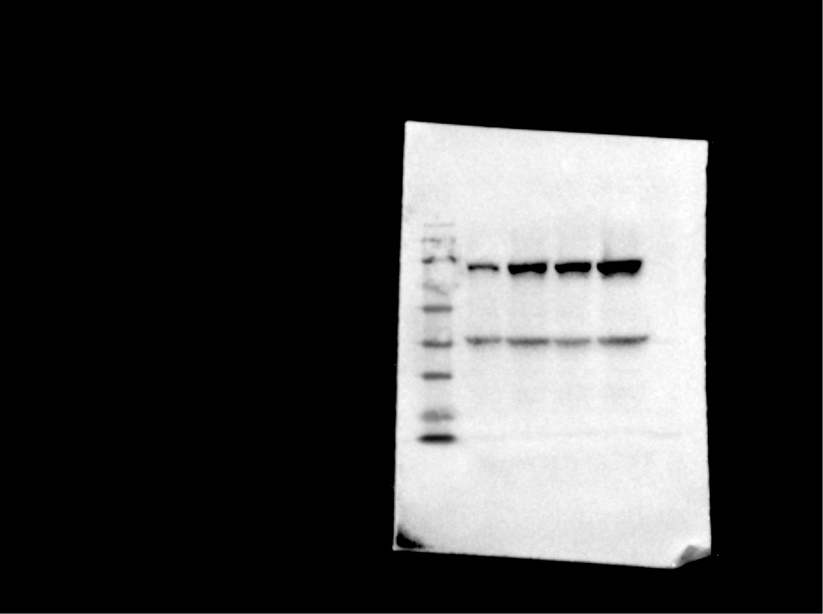


Figure 3E-5


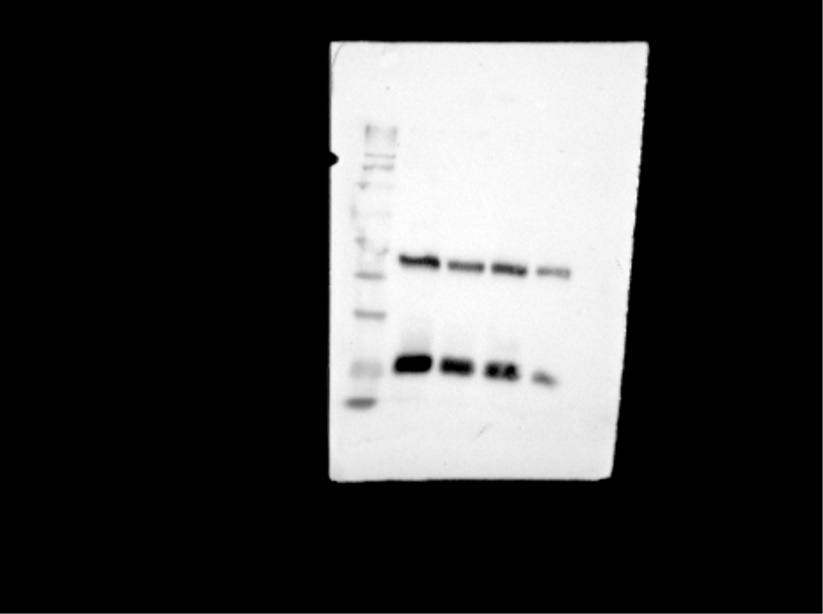


Figure 3E-6


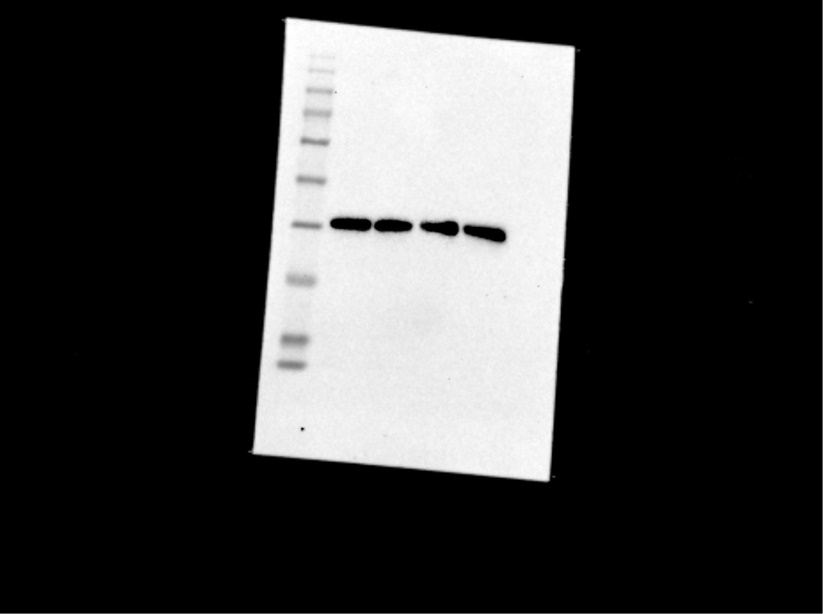


Figure 3E-7


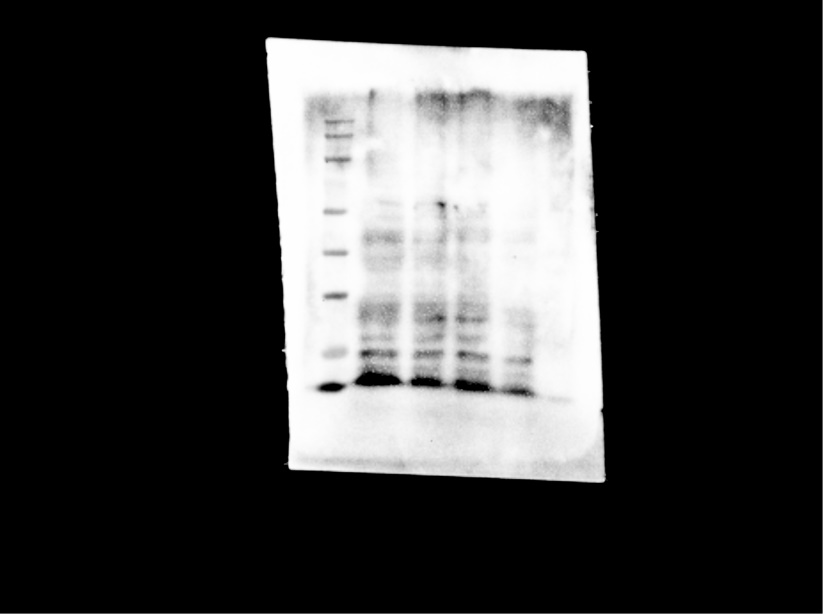


Figure 3F-1


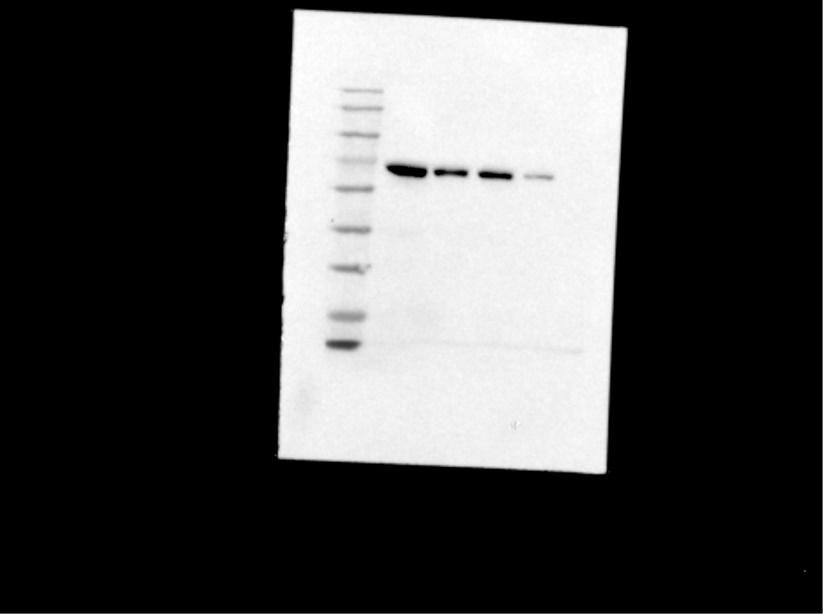


Figure 3F-2


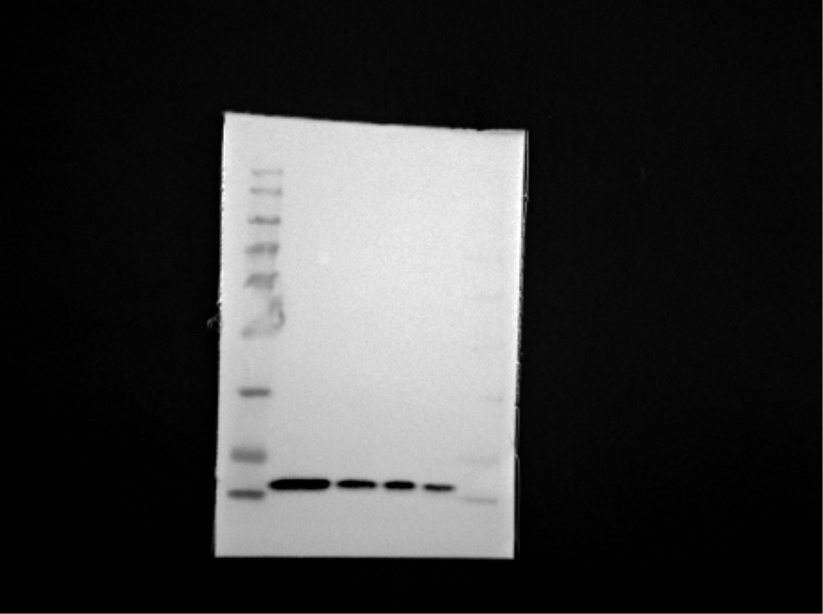


Figure 3F-3


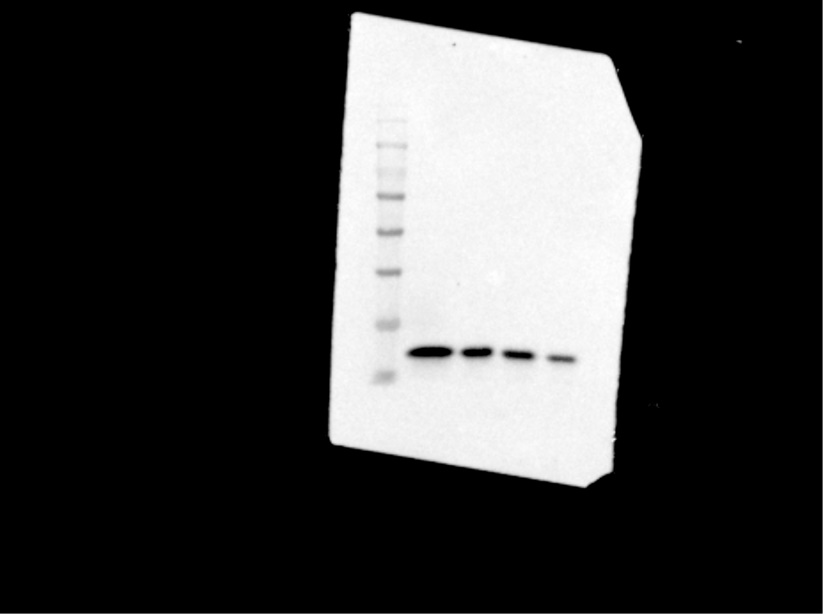


Figure 3F-4


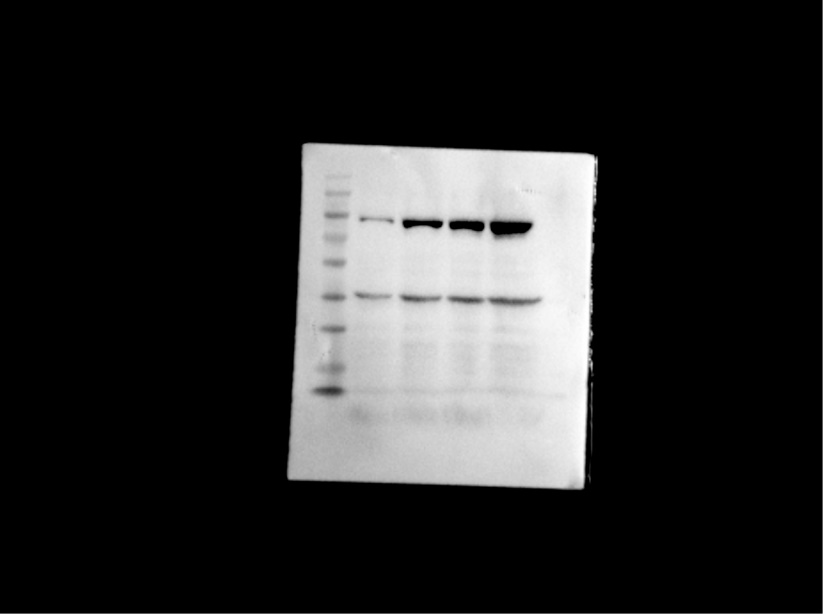


Figure 3F-5


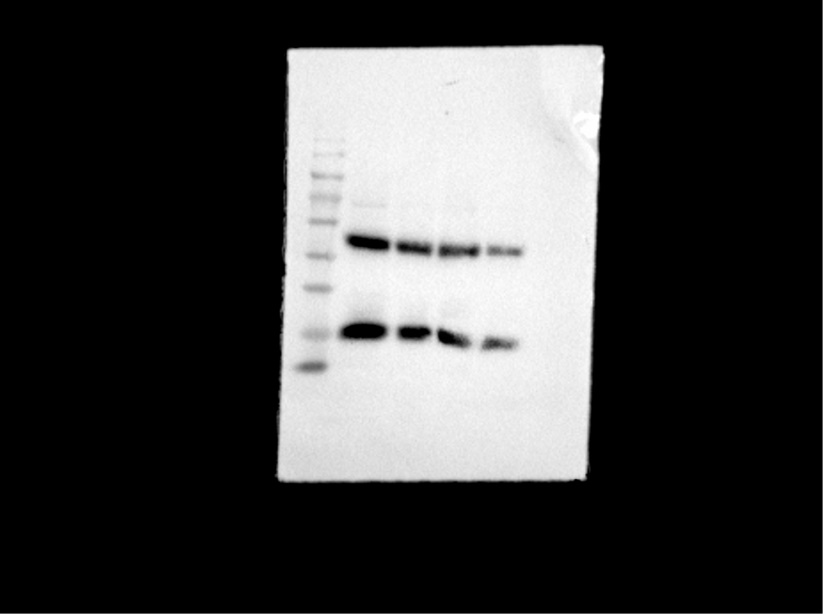


Figure 3F-6


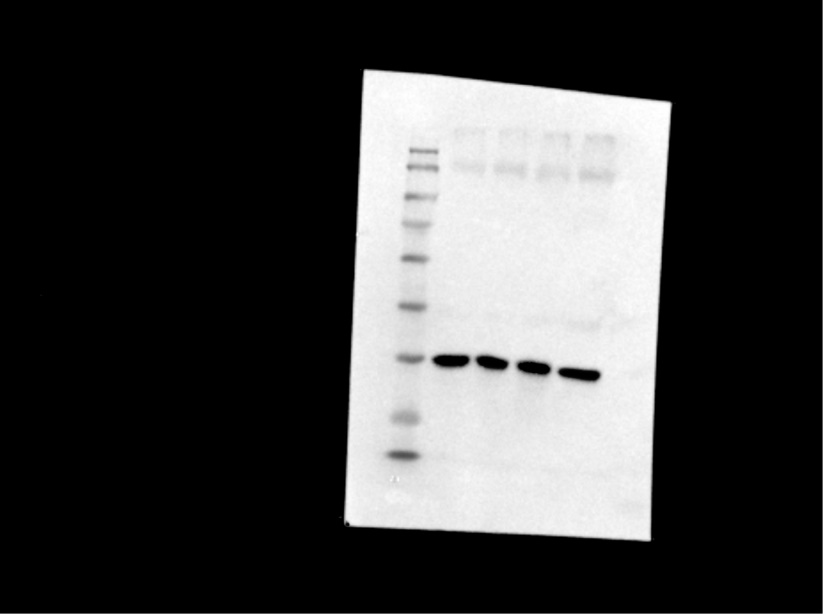


Figure 3F-7


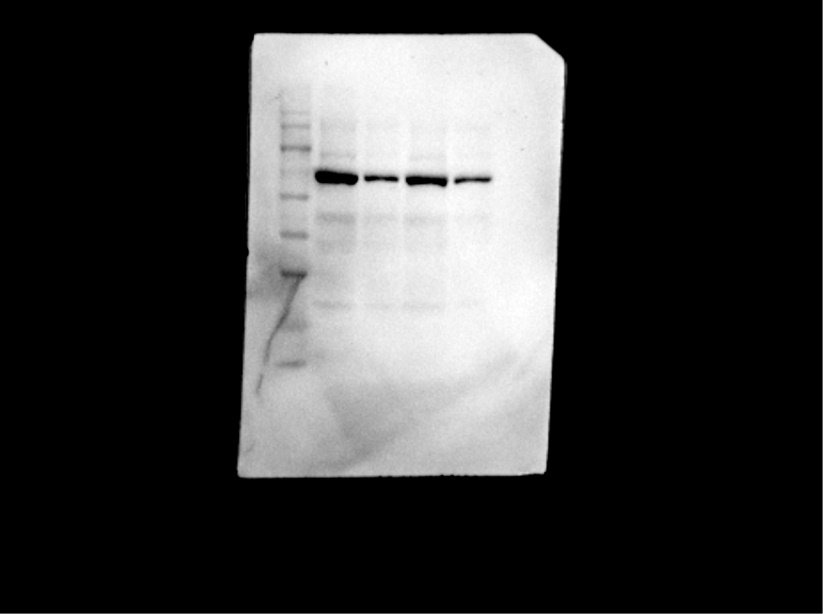


Figure 4D-1


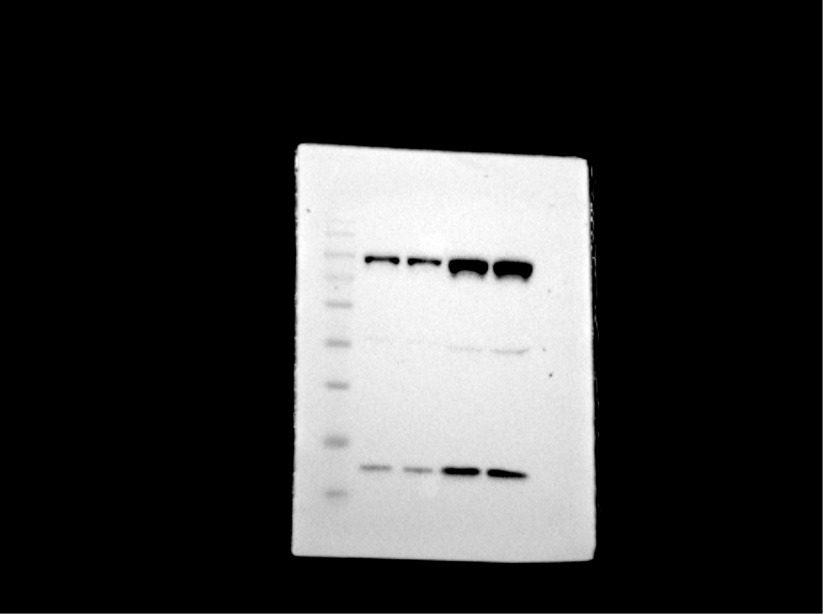


Figure 4D-2


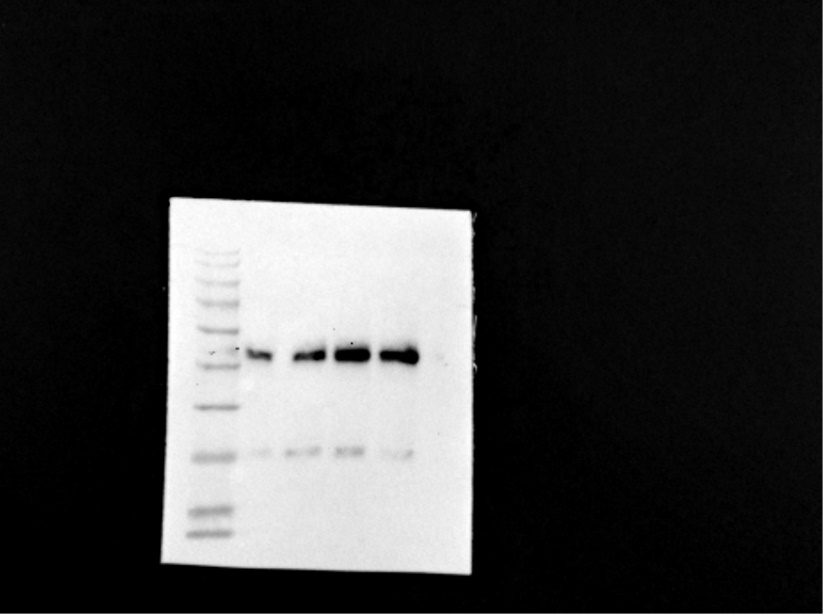


Figure 4D-3


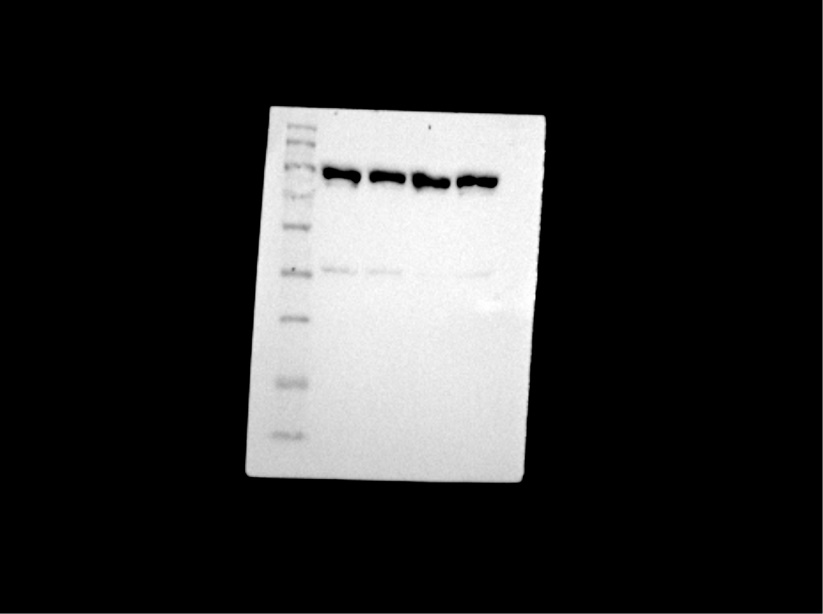


Figure 4D-4


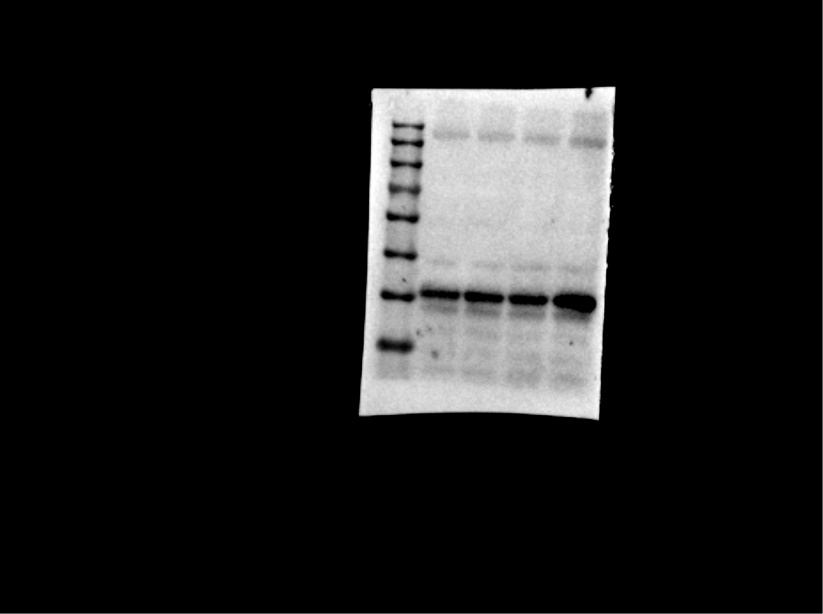


Figure 4D-5


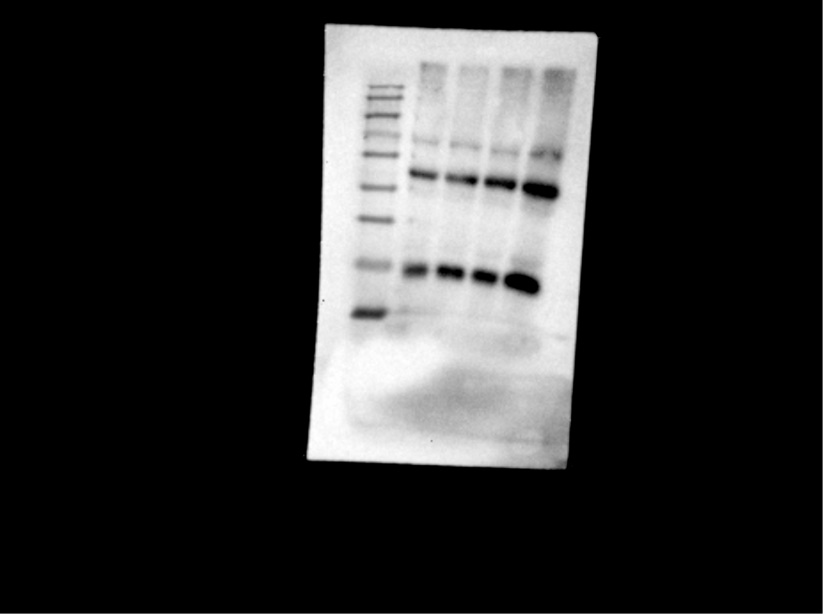


Figure 4D-6


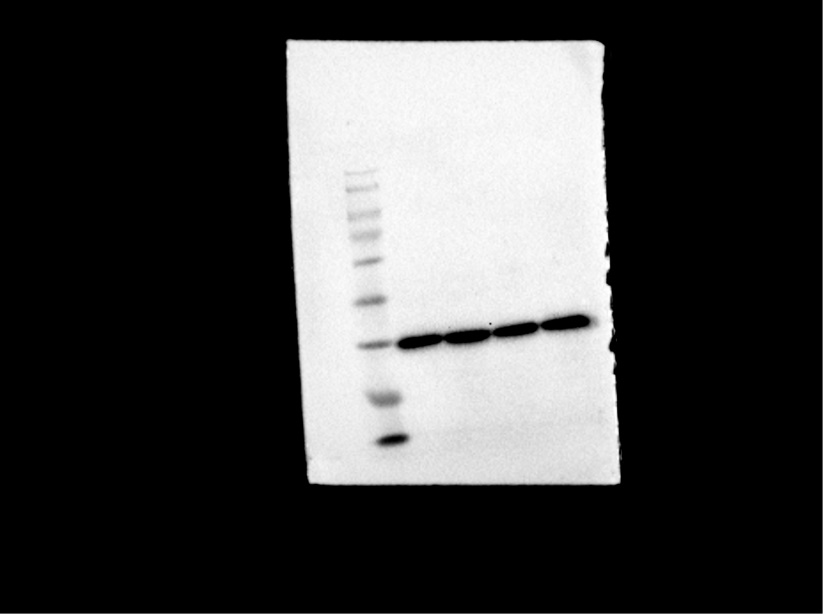


Figure 4D-7


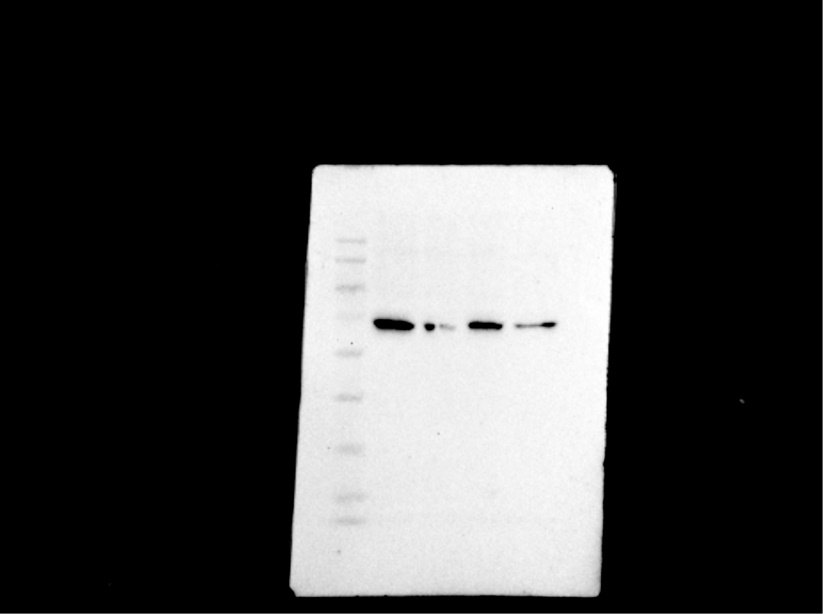


Figure 4E-1


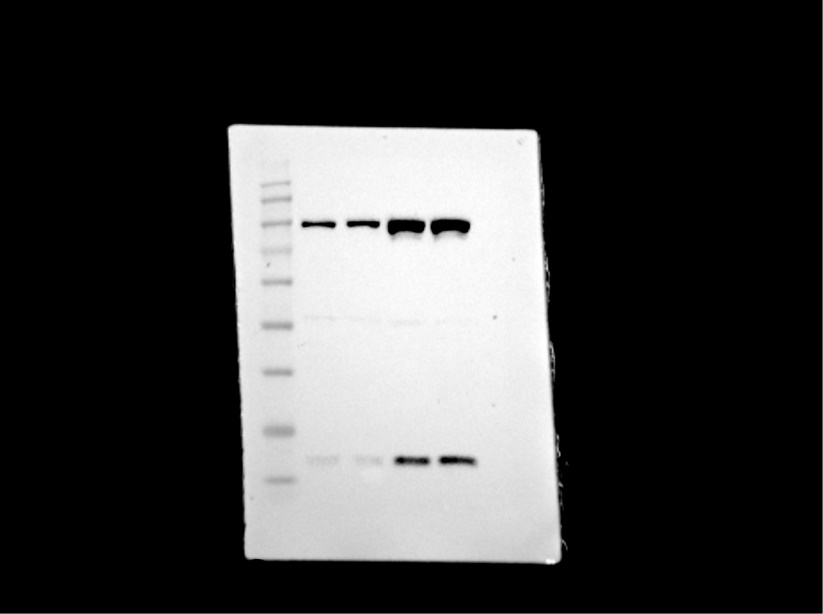


Figure 4E-2


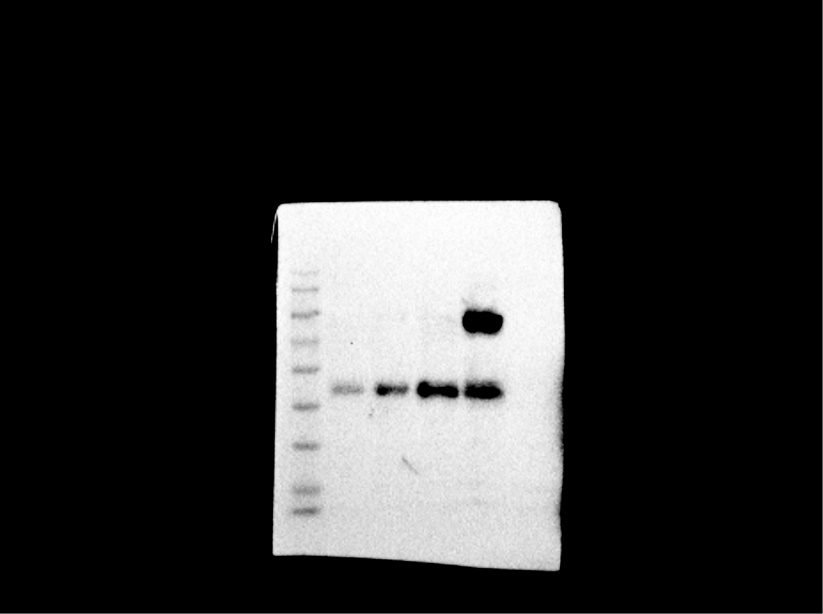


Figure 4E-3


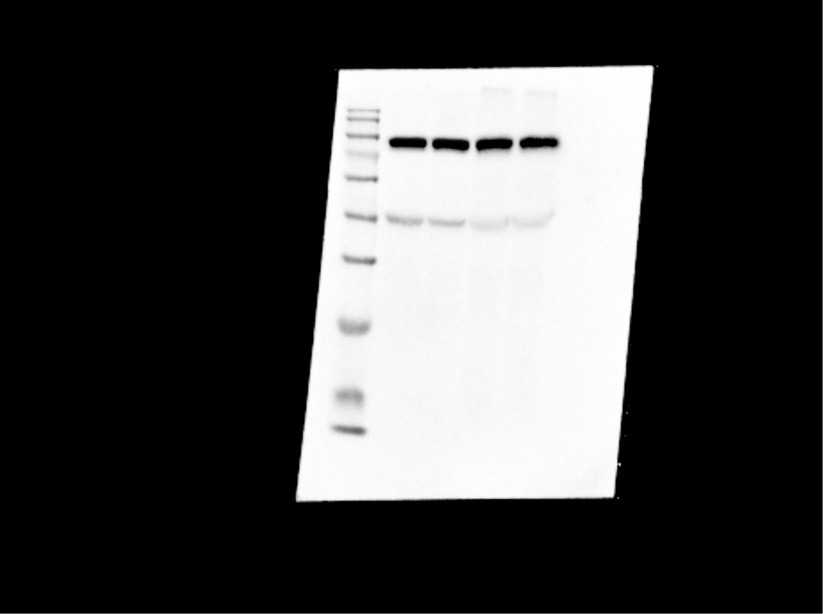


Figure 4E-4


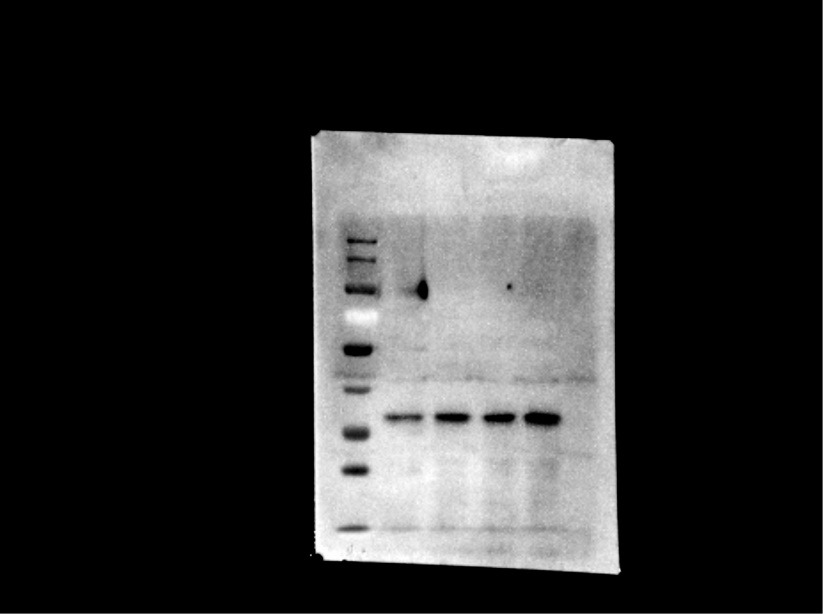


Figure 4E-5


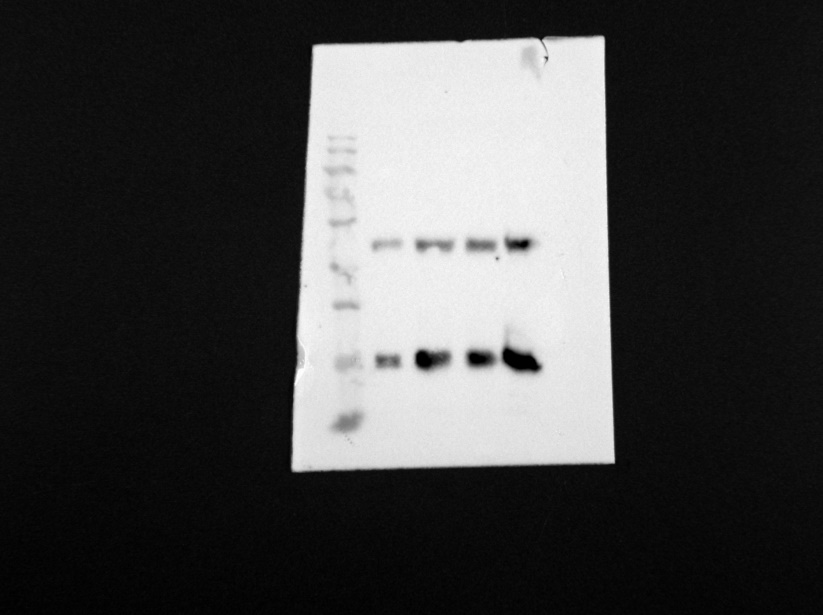


Figure 4E-6


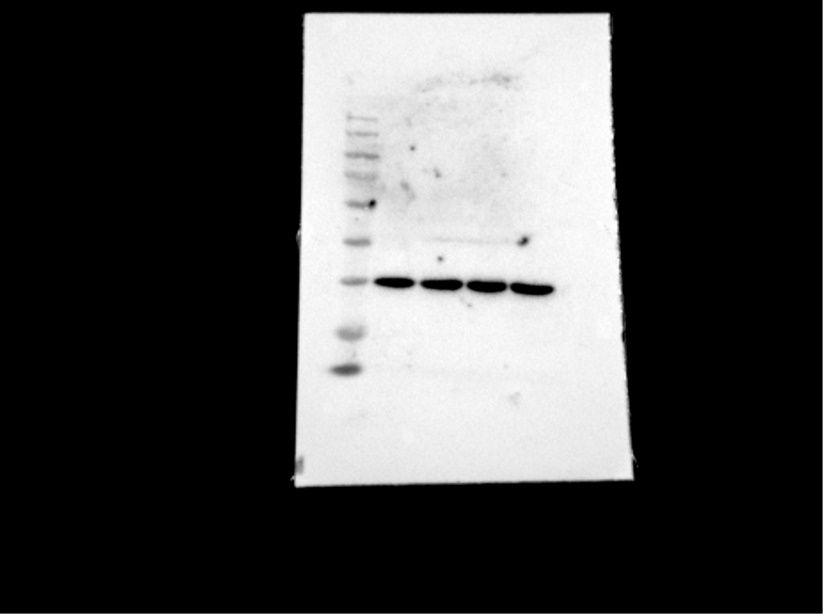


Figure 4D-7


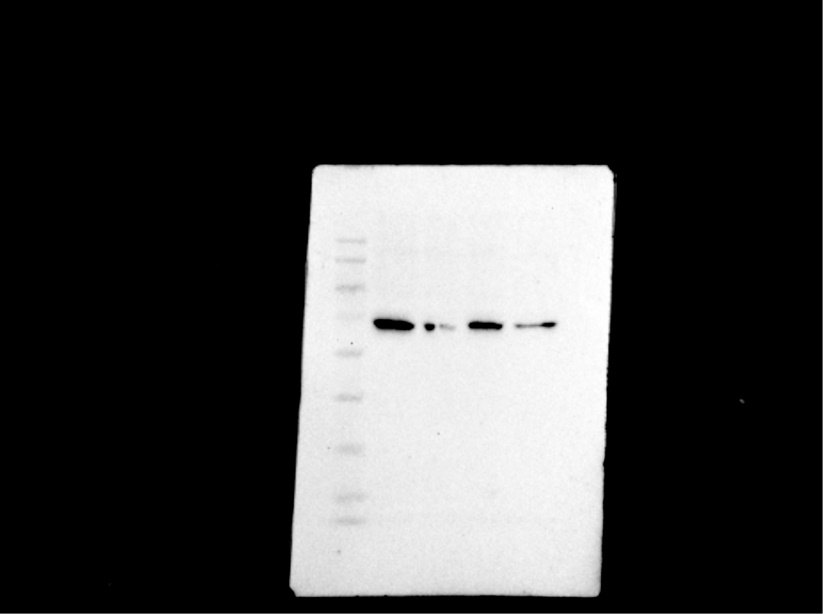


Figure 4E-1


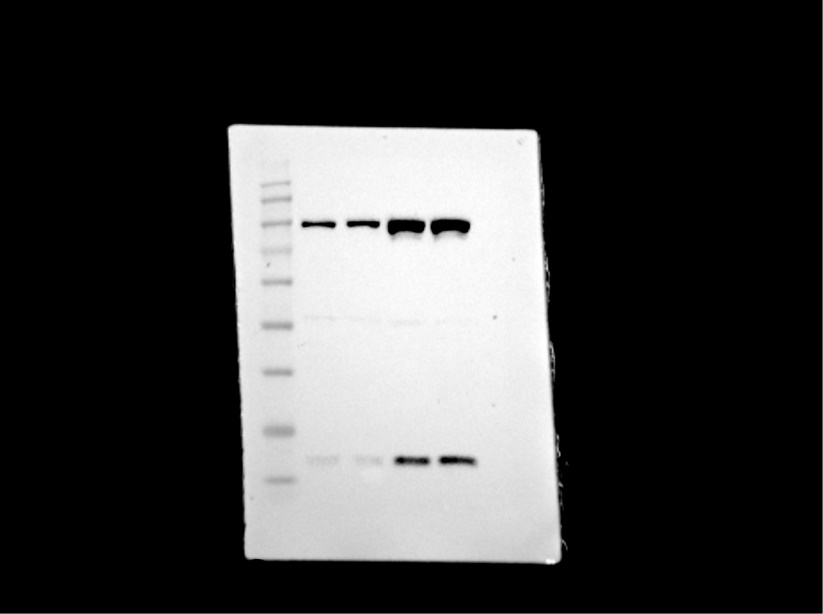


Figure 4E-2


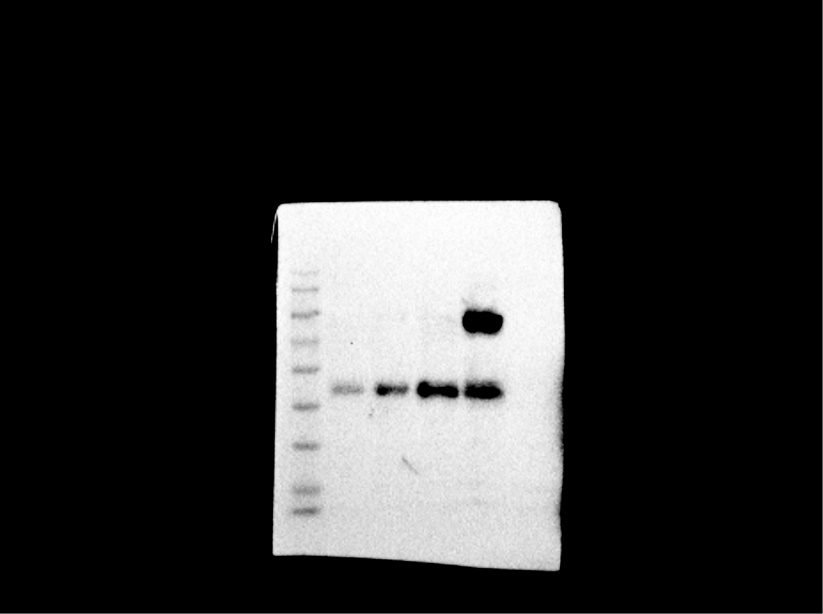


Figure 4E-3


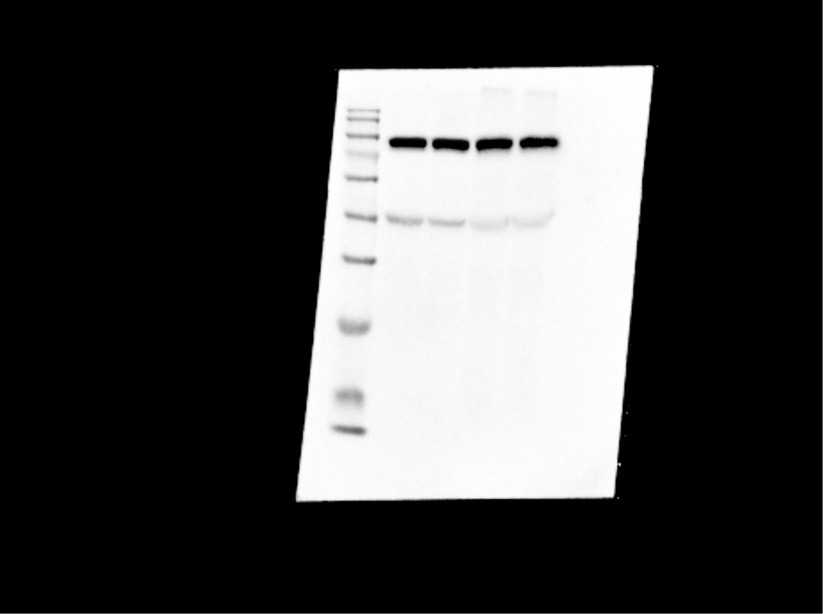


Figure 4E-4


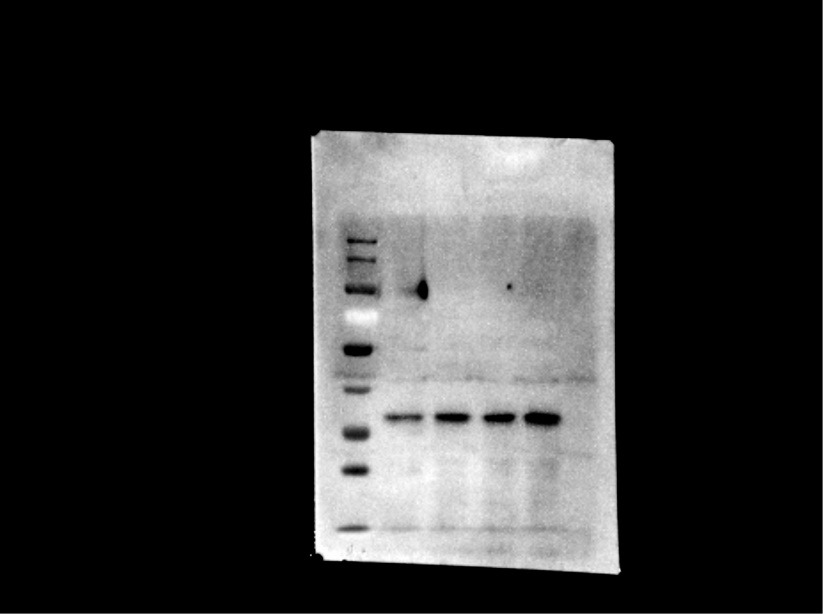


Figure 4E-5


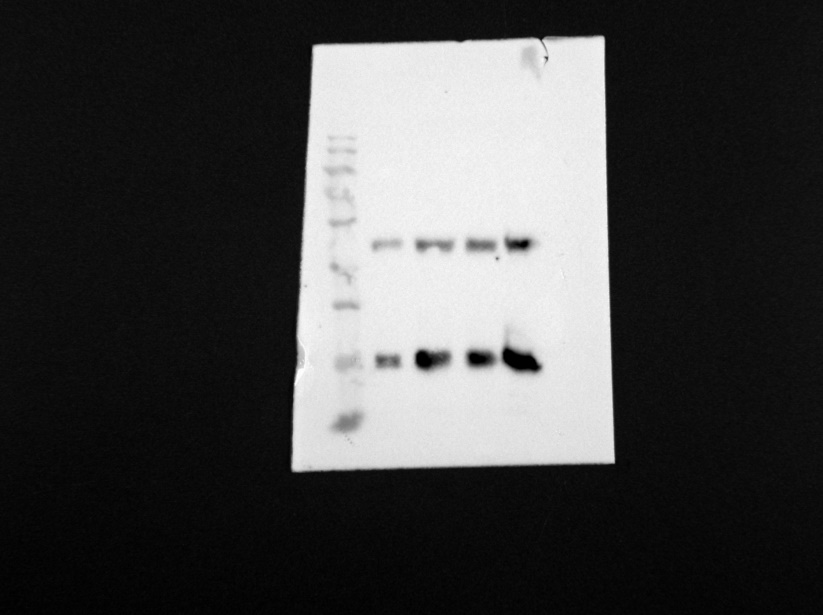


Figure 4E-6


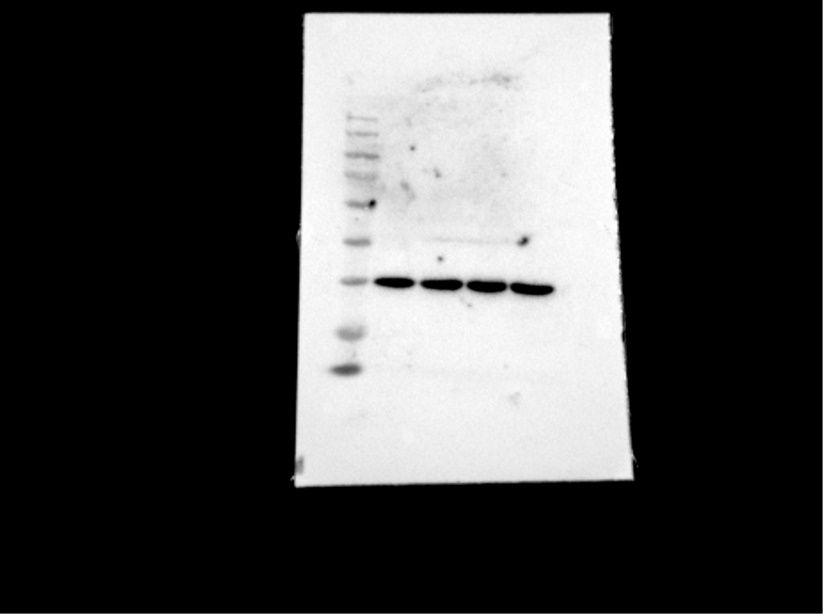


Figure 4E-7


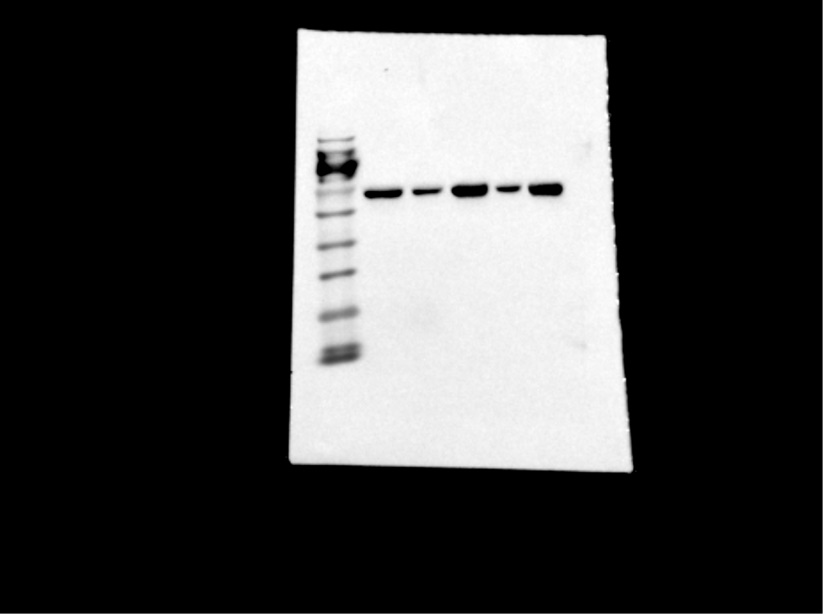


Figure 5I-1


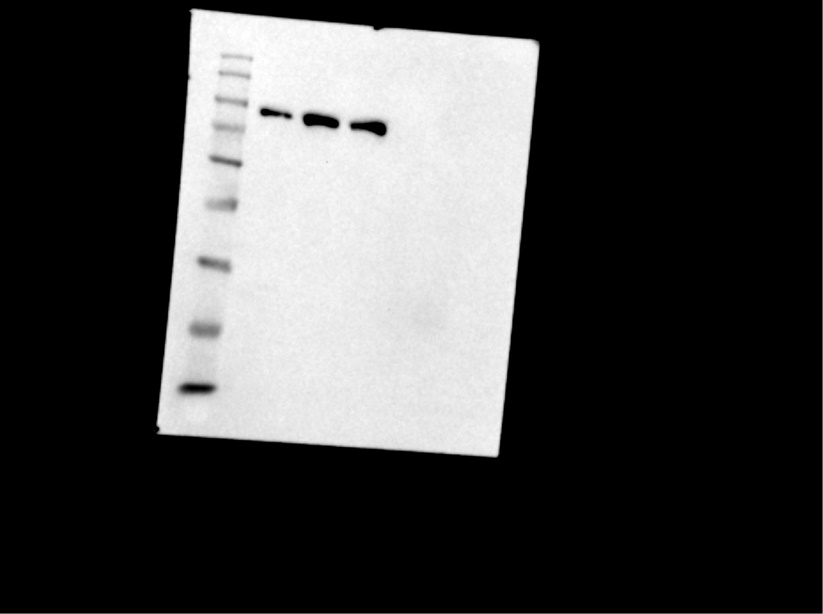


Figure 5I-2


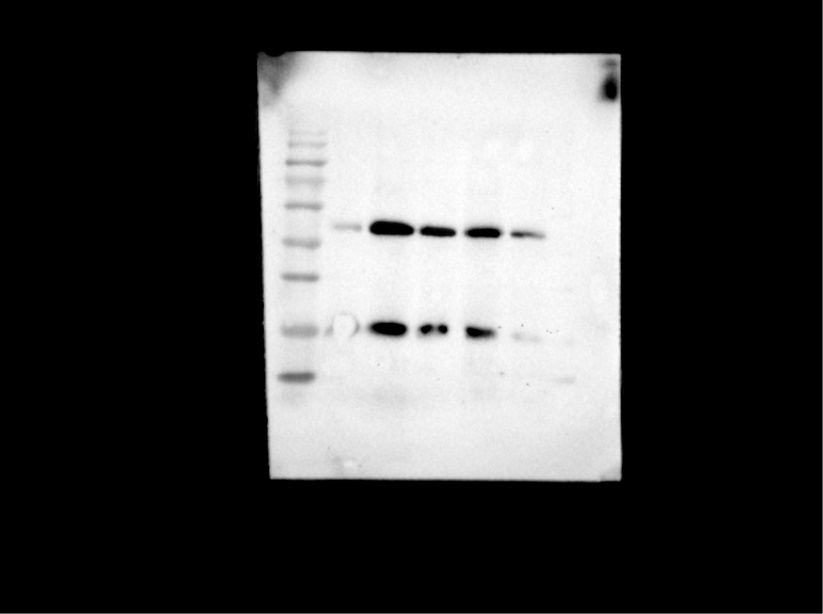


Figure 5I-3


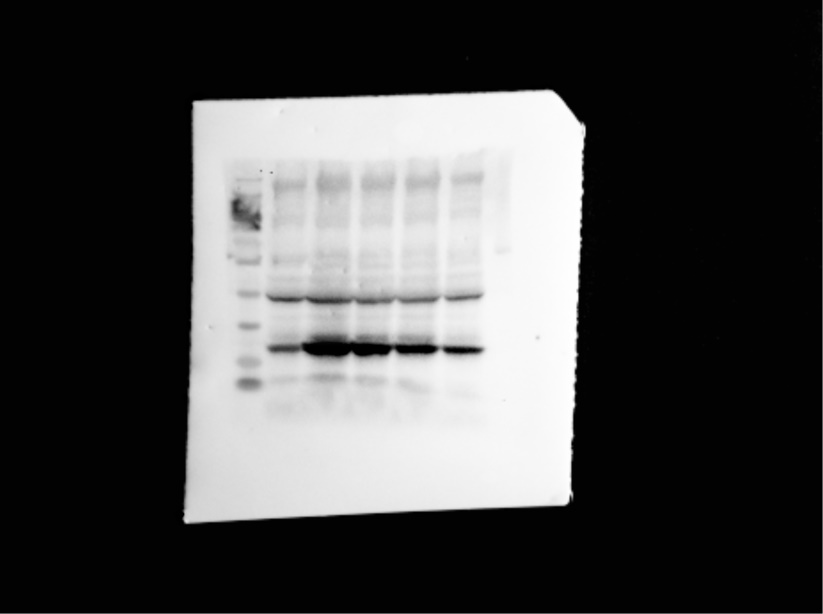


Figure 5I-4


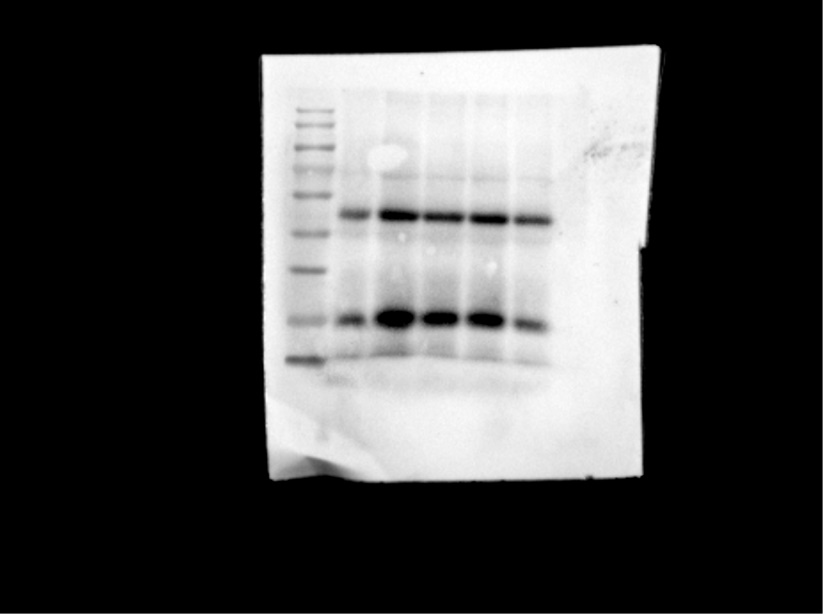


Figure 5I-5


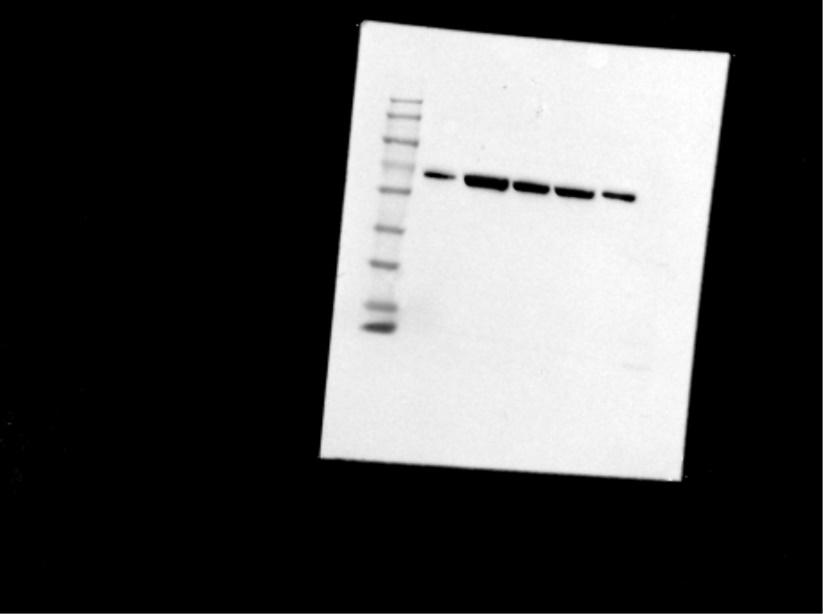


Figure 5I-6


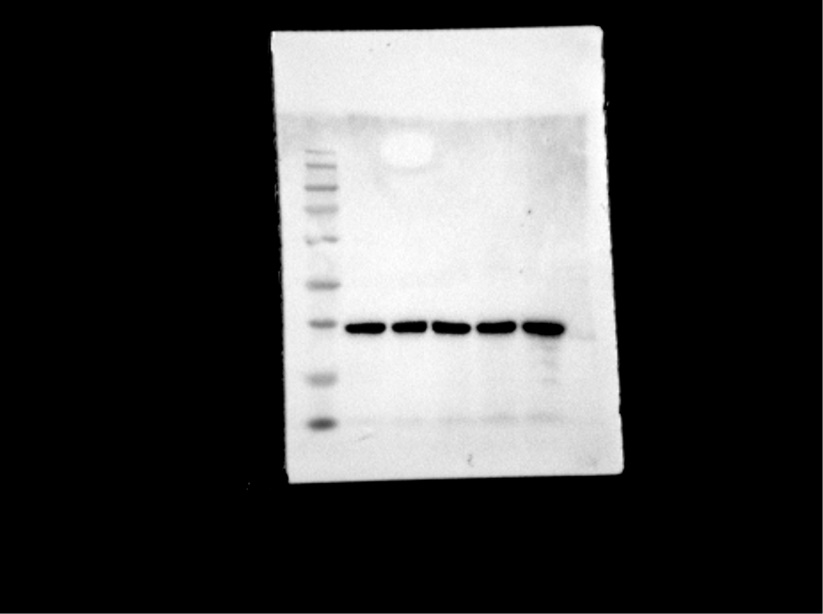


Figure 5I-7


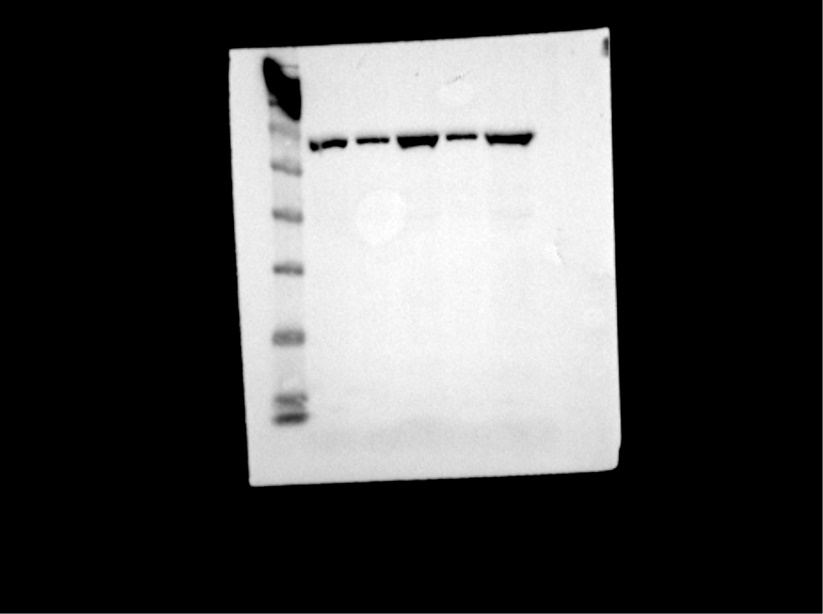


Figure 6H-1


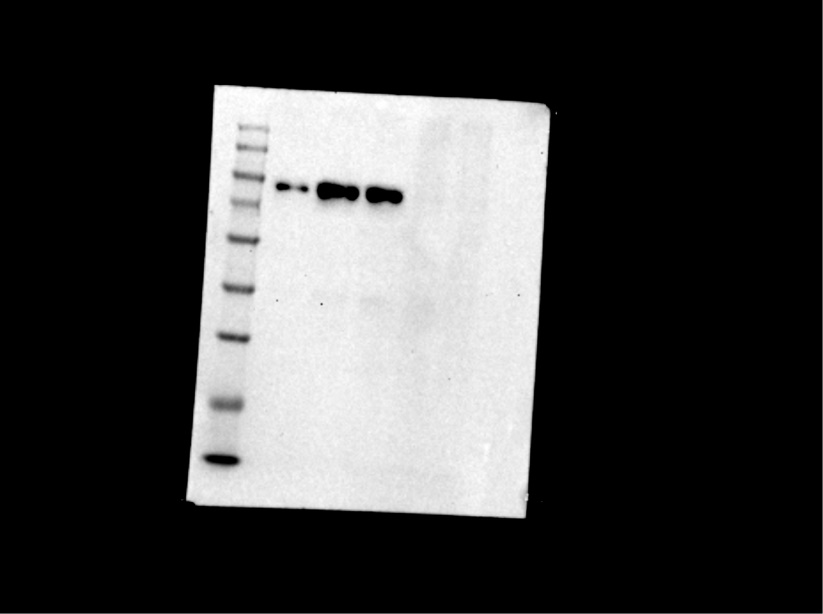


Figure 6H-2


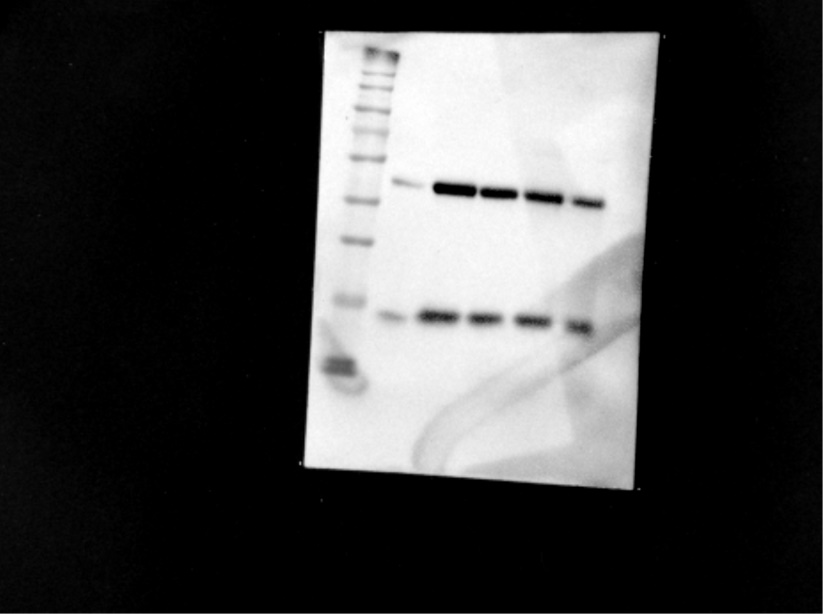


Figure 6H-3


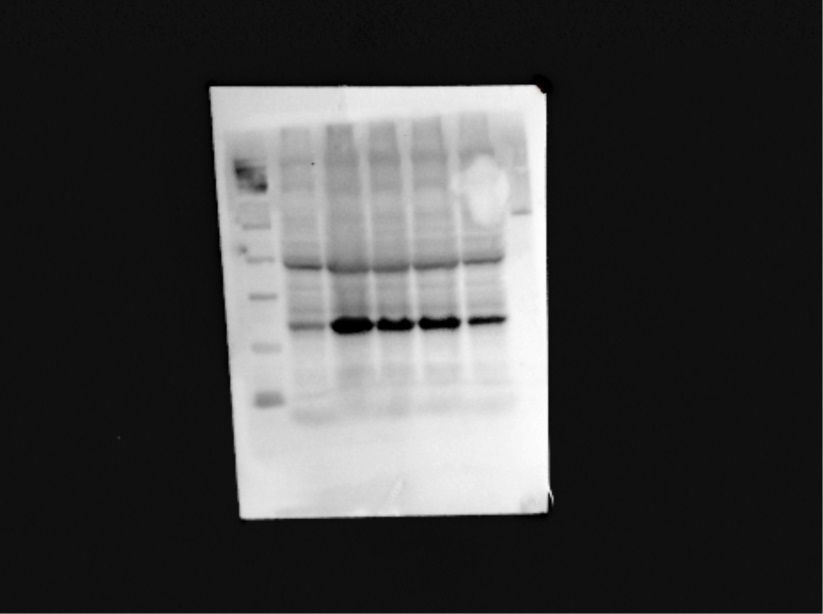


Figure 6H-4


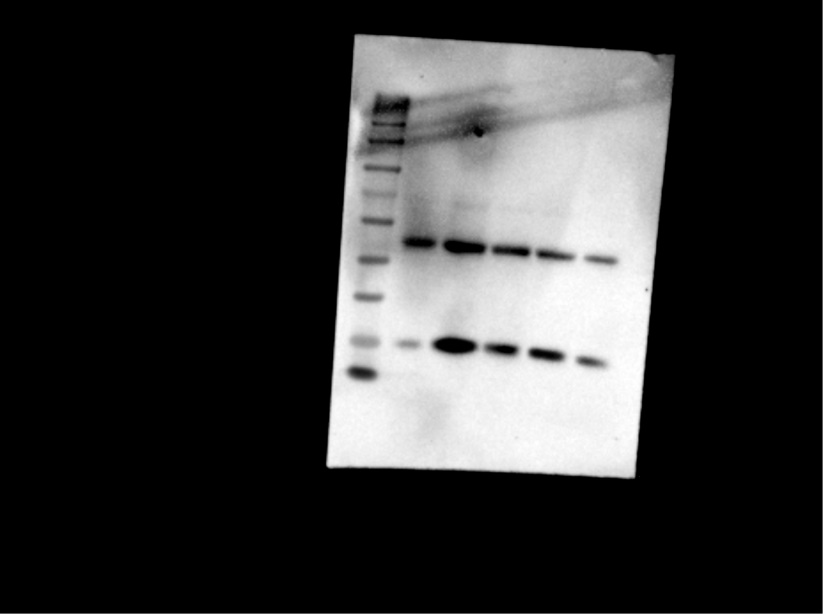


Figure 6H-5


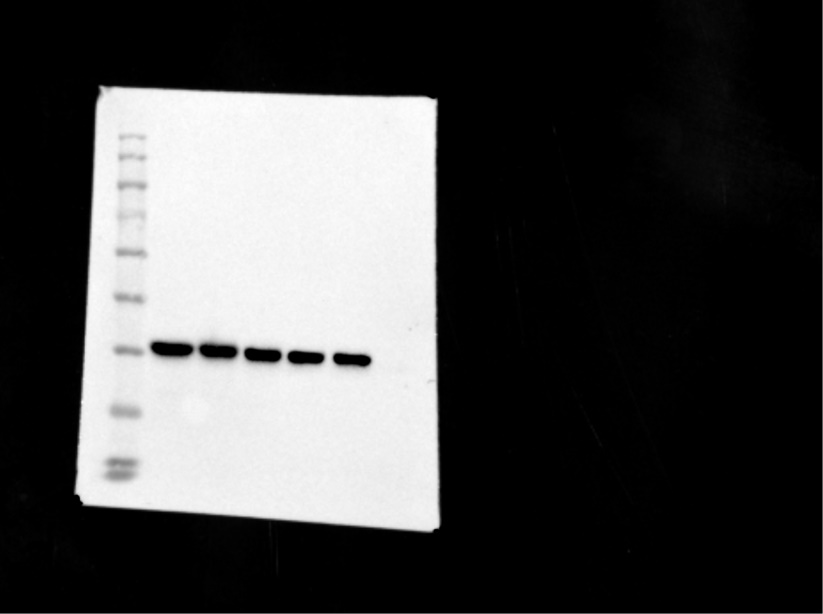


Figure 6H-6


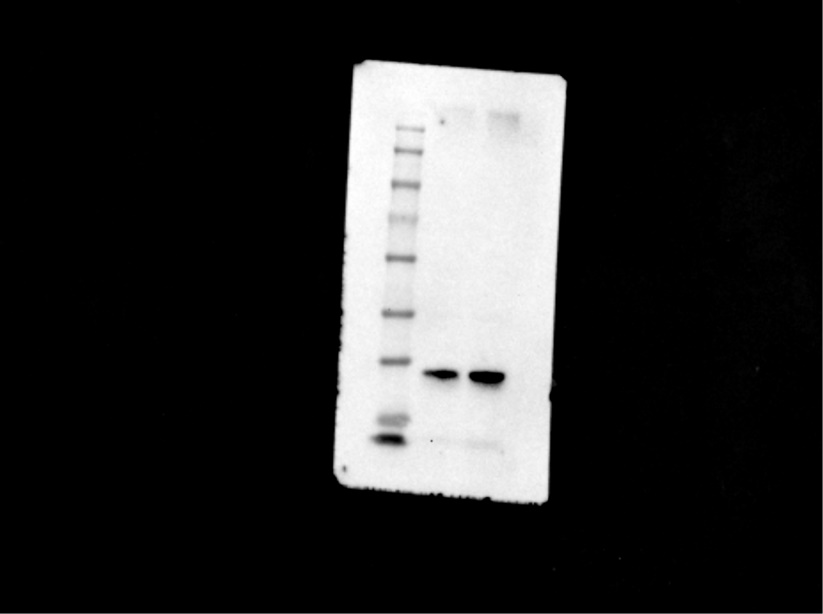


Figure S5G-1


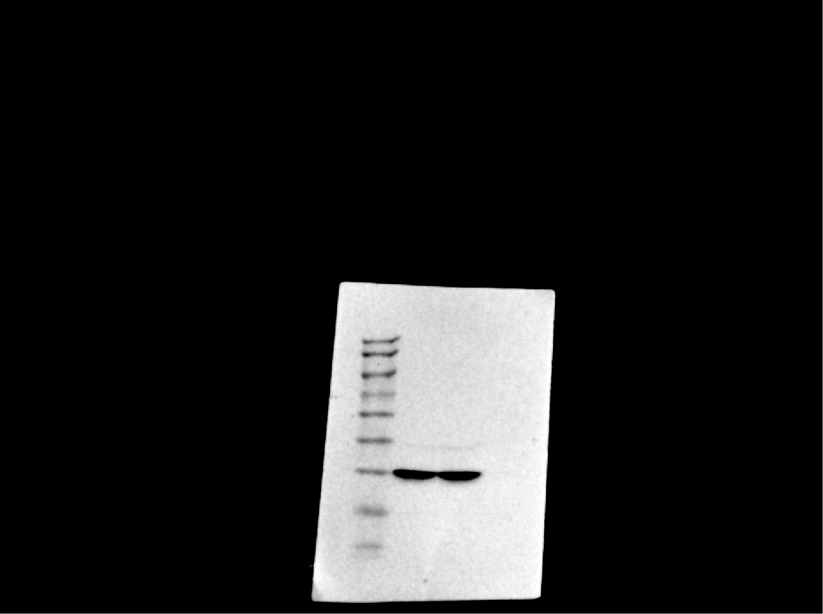


Figure S5G-2


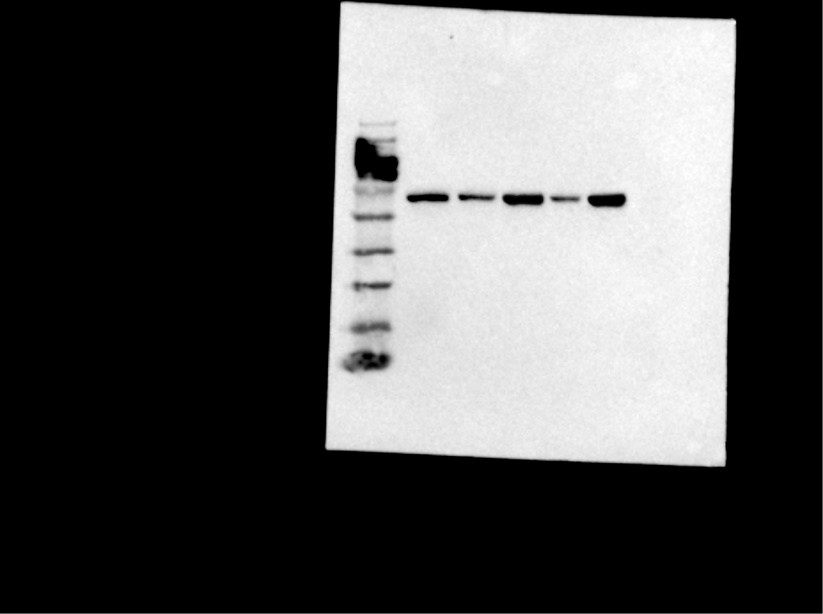


Figure S6I-1


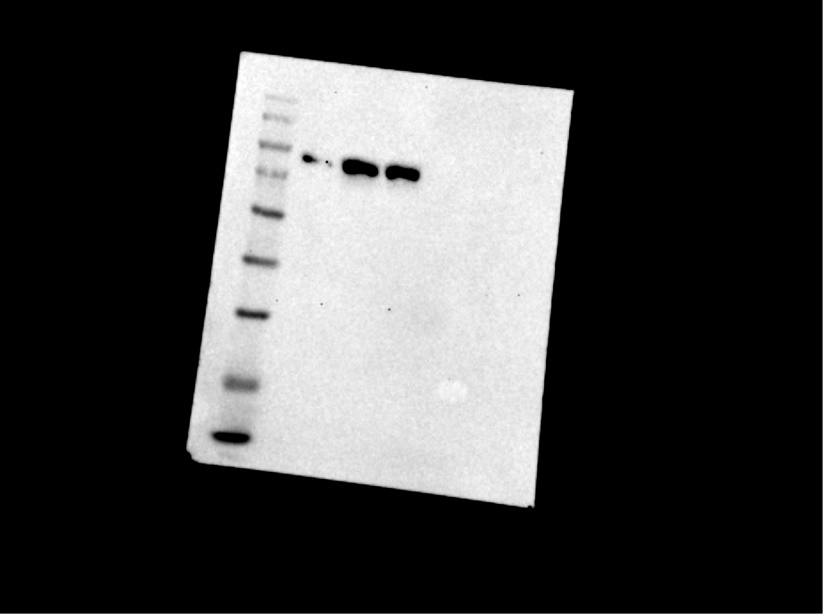


Figure S6I-2


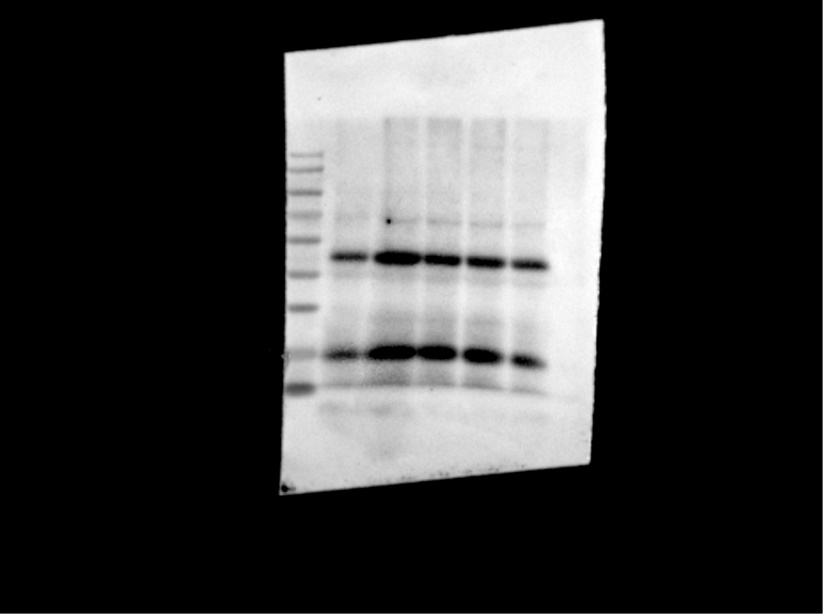


Figure S6I-3


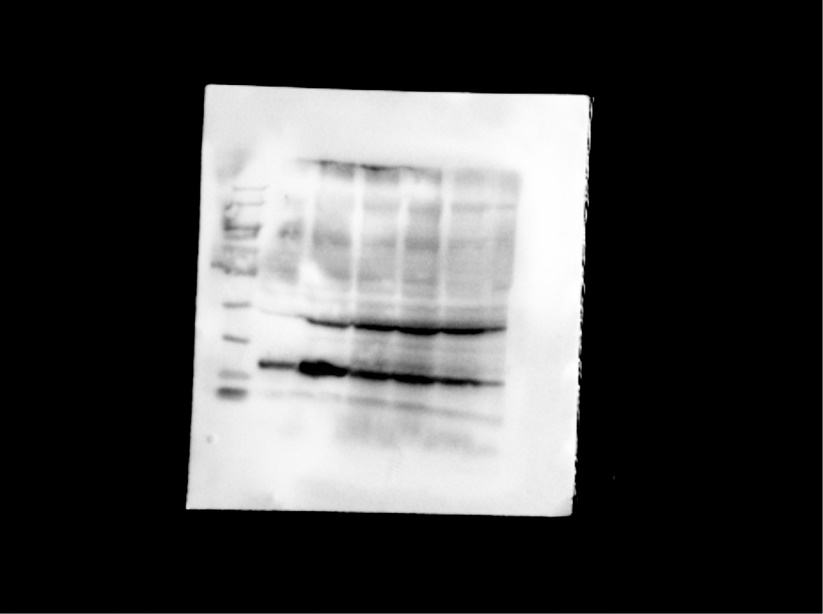


Figure S6I-4


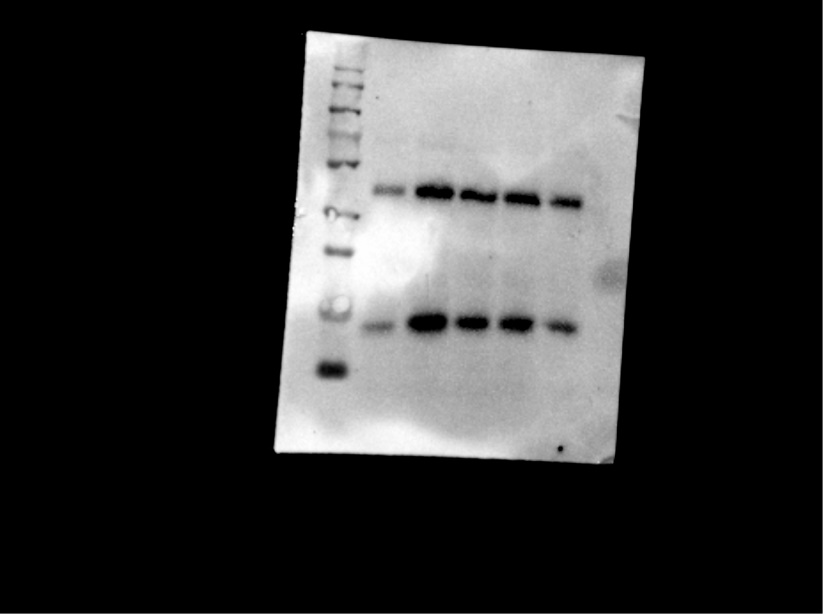


Figure S6I-5


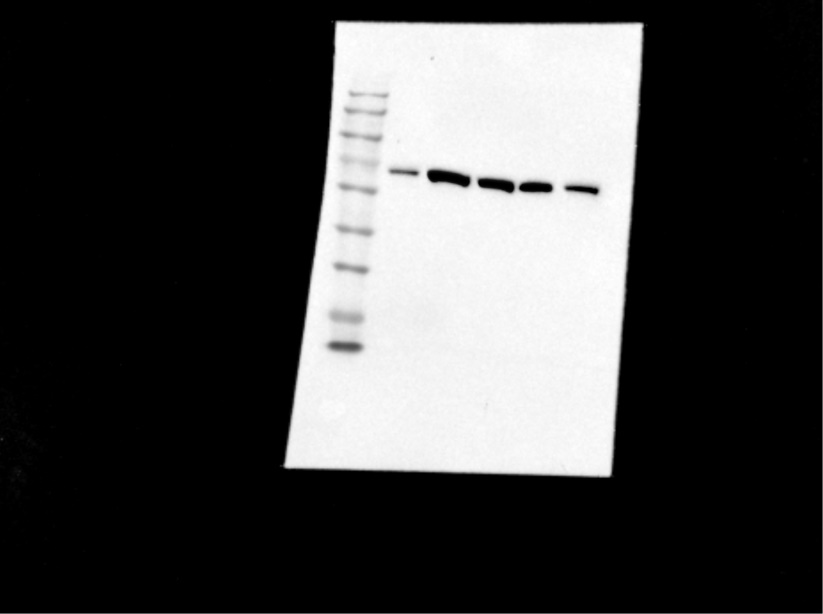


Figure S6I-6


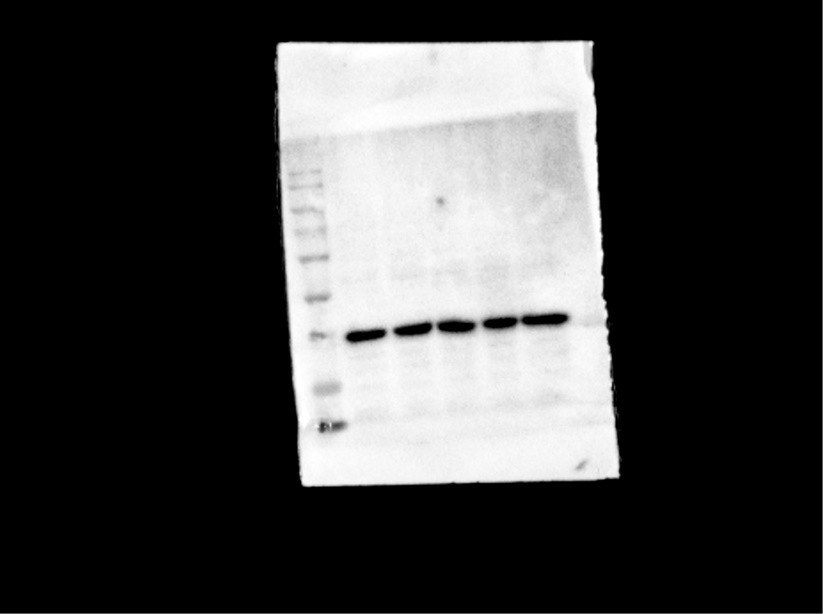


Figure S6I-7


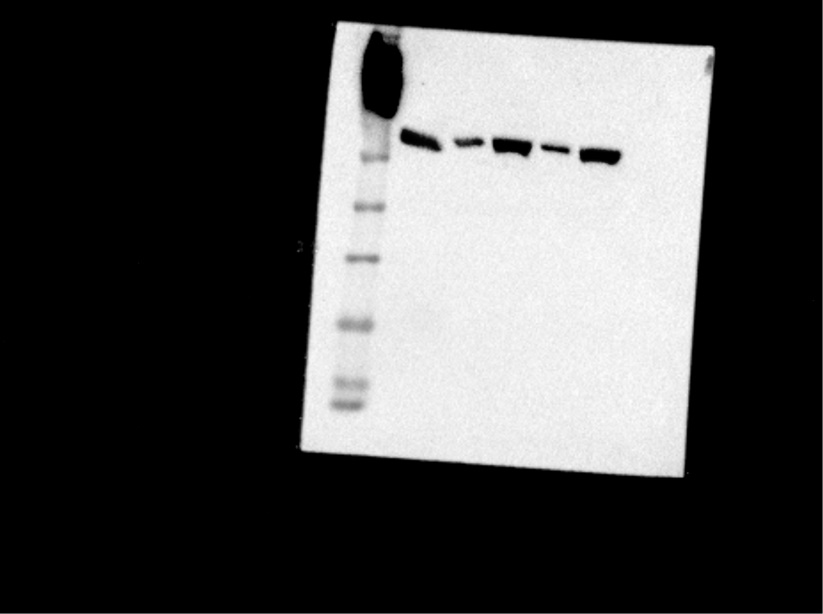


Figure S7H-1


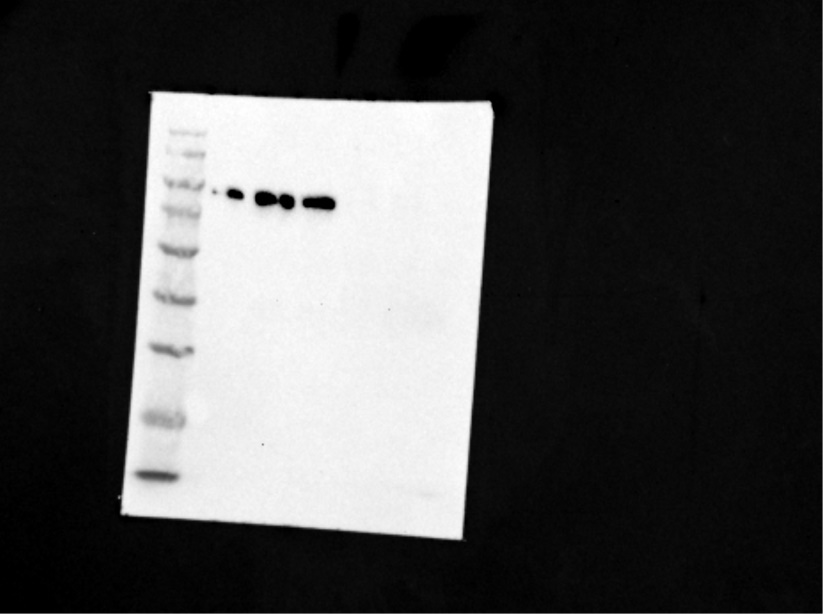


Figure S7H-2


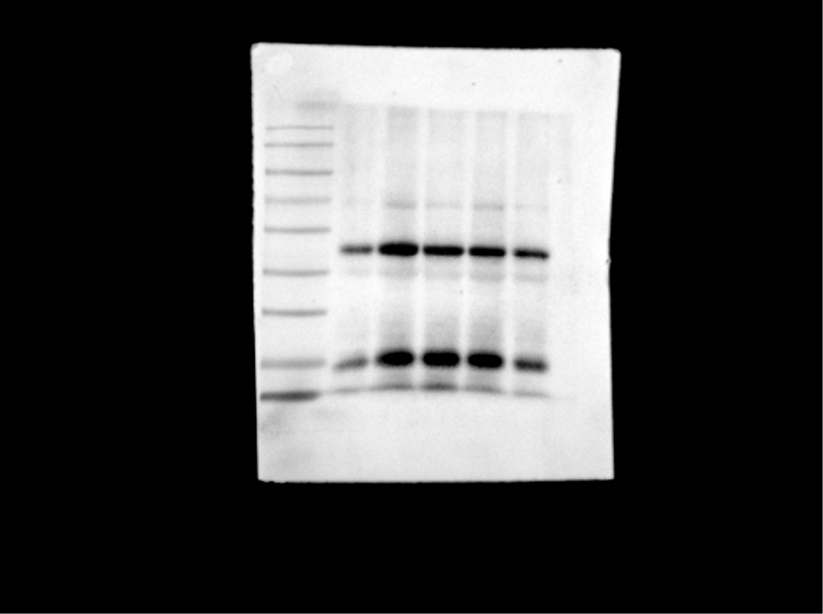


Figure S7H-3


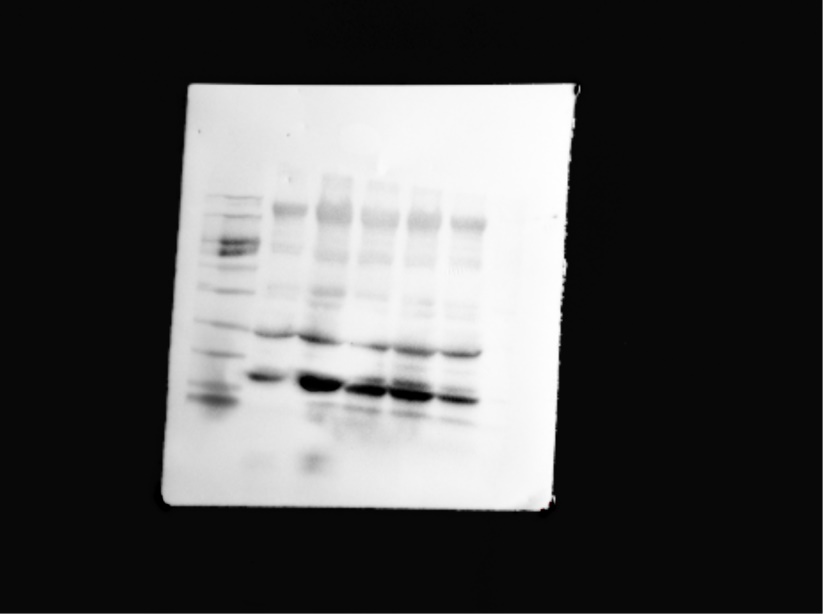


Figure S7H-4


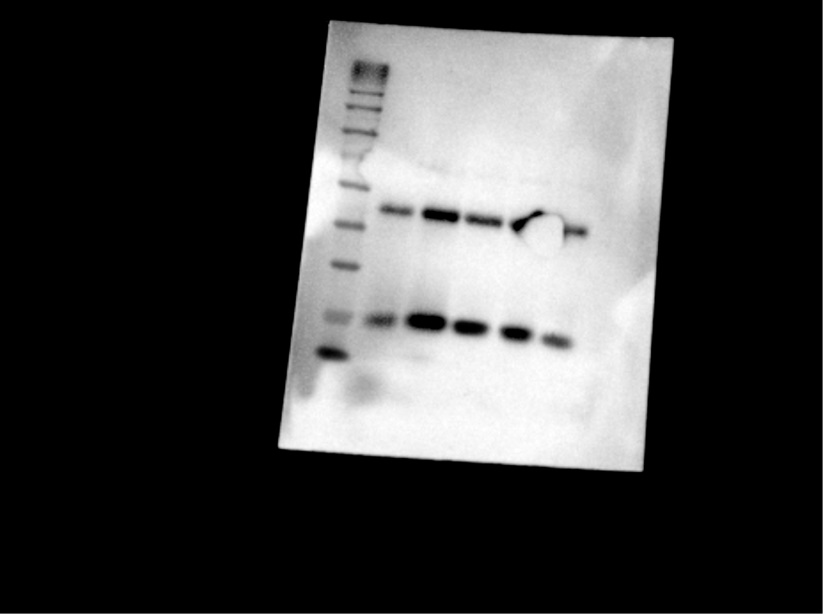


Figure S7H-5


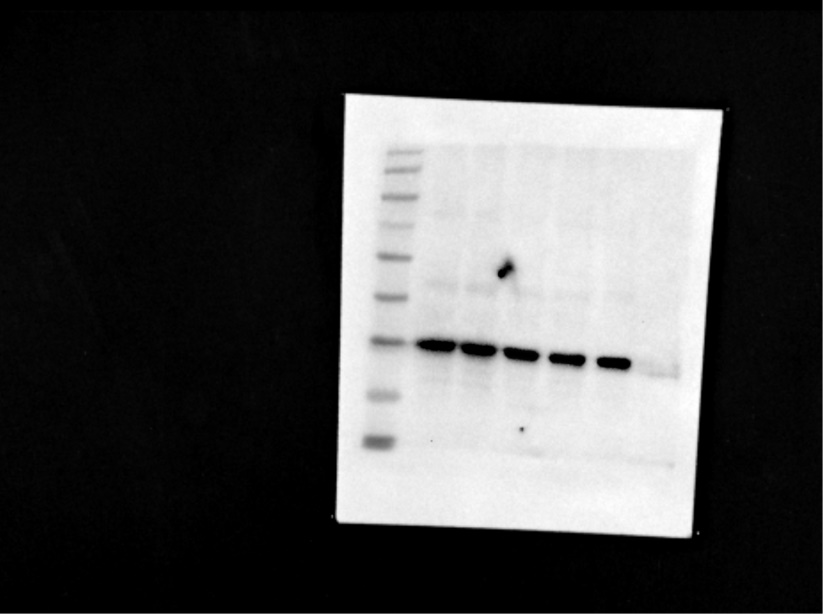


Figure S7H-6
