# Supplementary figures and images for: Meta‐analysis and transcriptomic analysis reveal that NKRF and ZBTB17 regulate the NF‐κB signaling pathway, contributing to the shared molecular mechanisms of Alzheimer's disease and atherosclerosis
Source: CNS Neurosci Ther. 2024 May 13;30(5):e14683. doi: 10.1111/cns.14683 (PMC11090078; doi:10.1111/cns.14683)

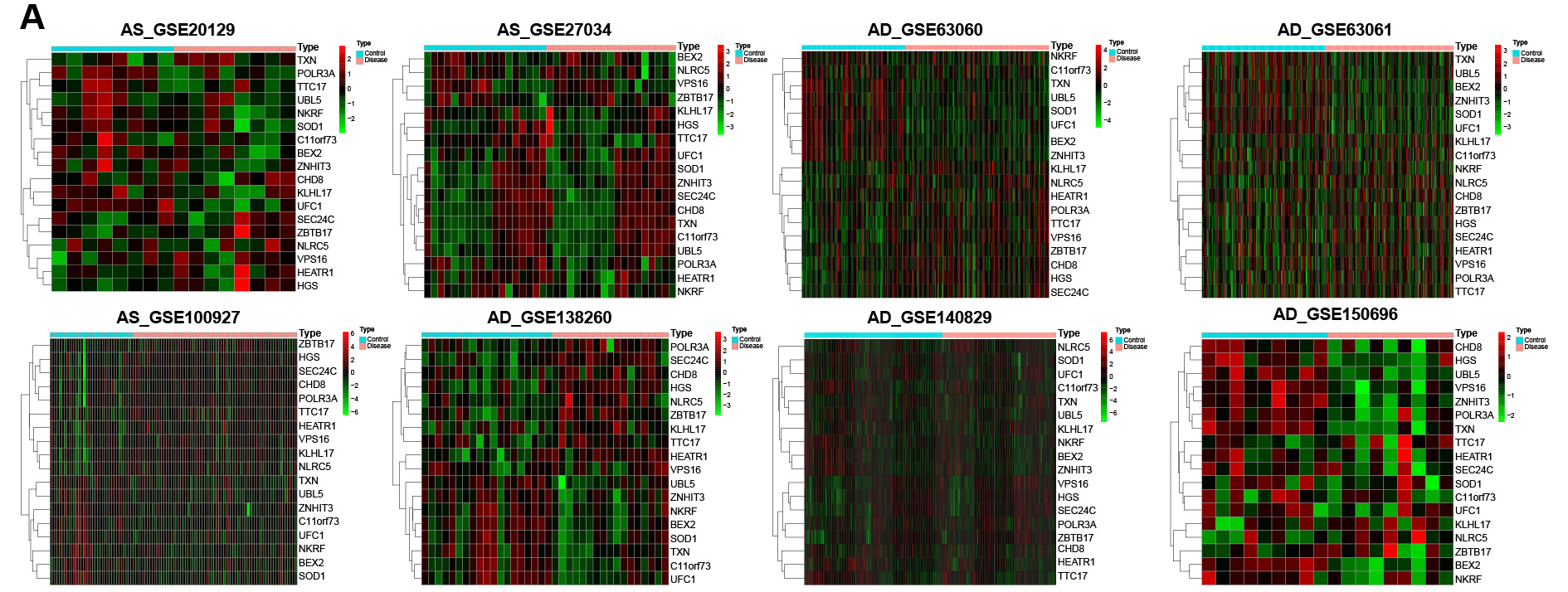

Supplement: Supplementary file 2 — Figure S1. [file CNS-30-e14683-s009.jpg]

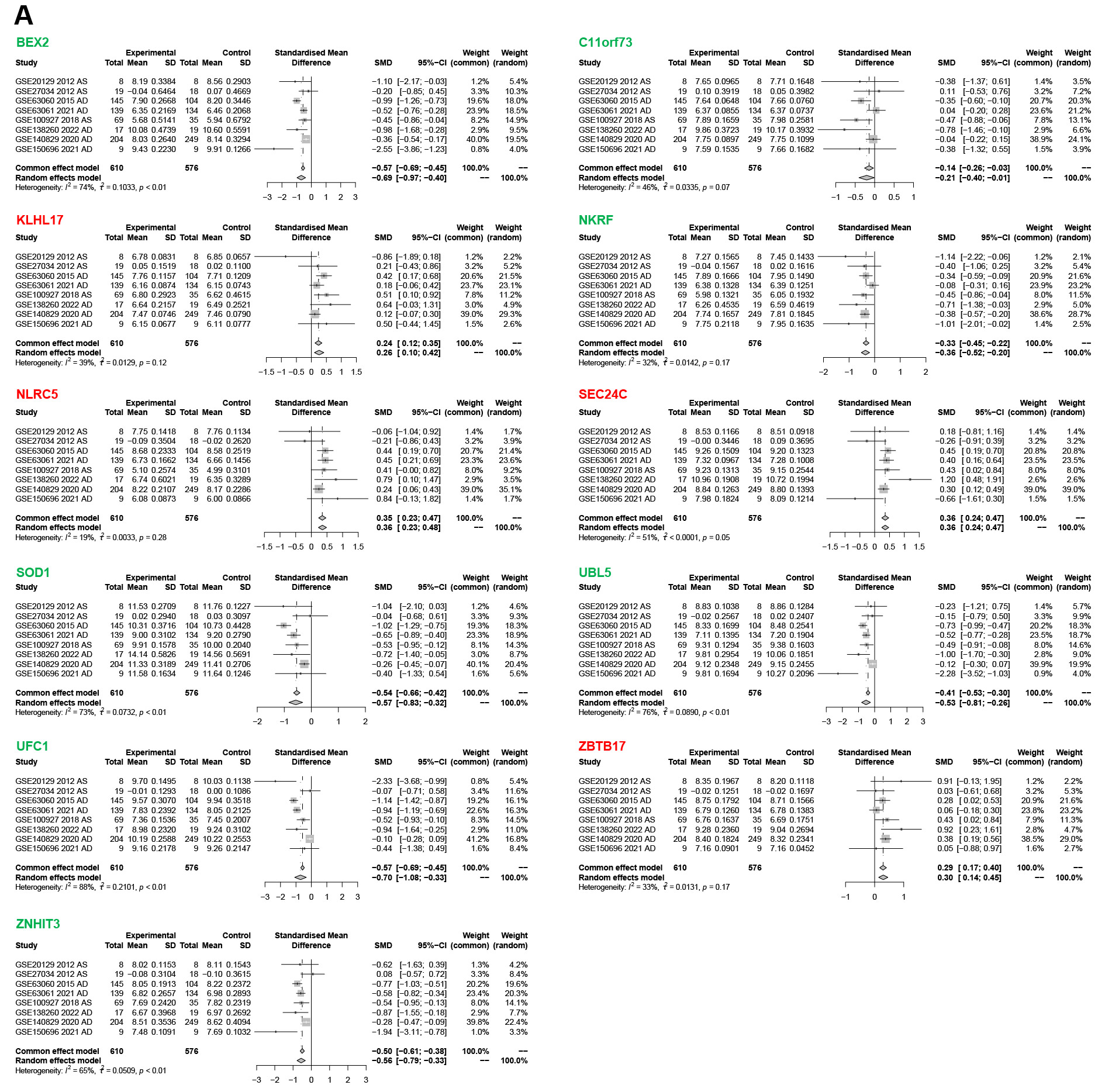

Supplement: Supplementary file 3 — Figure S2. [file CNS-30-e14683-s006.jpg]

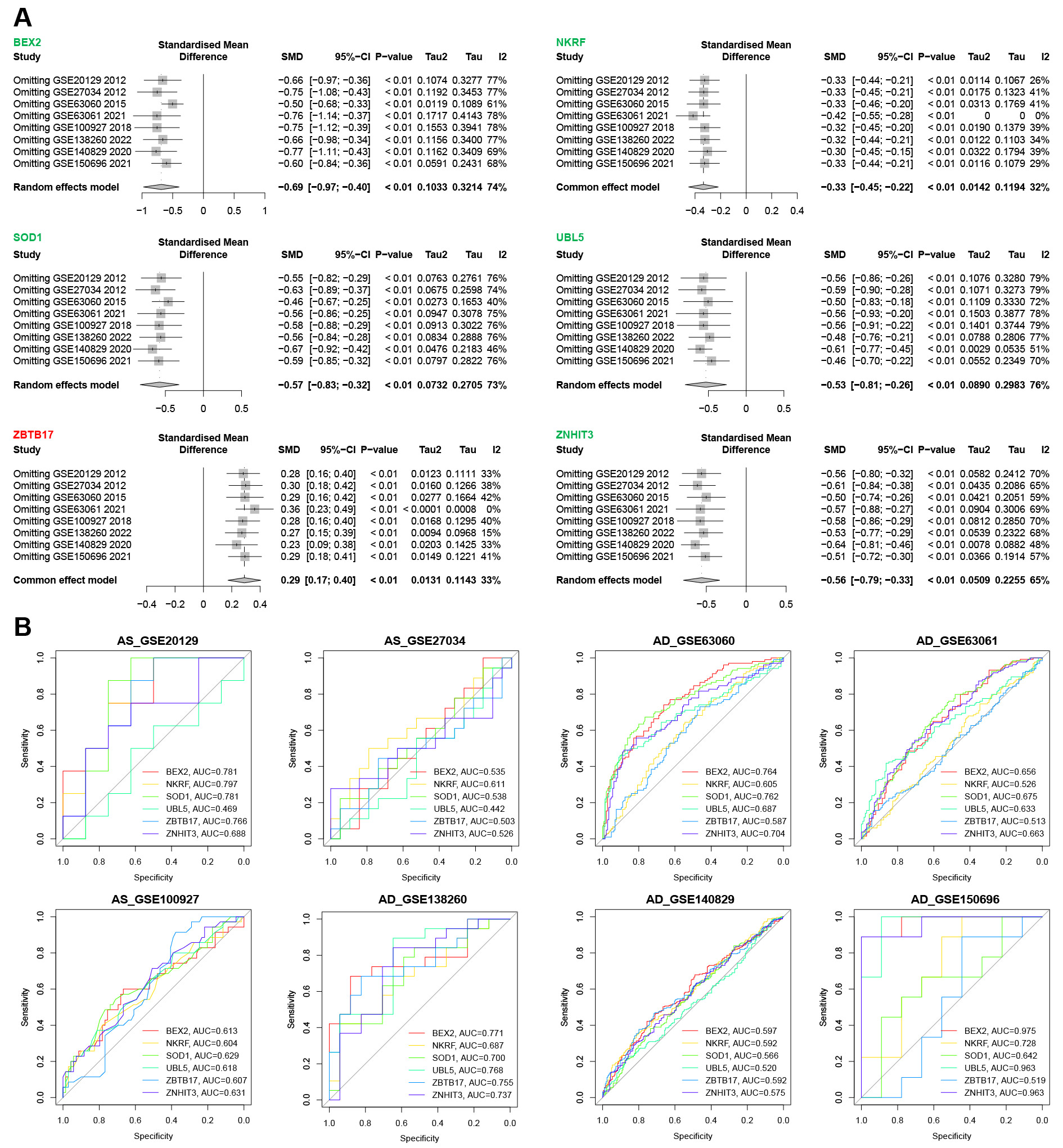

Supplement: Supplementary file 4 — Figure S3. [file CNS-30-e14683-s008.jpg]

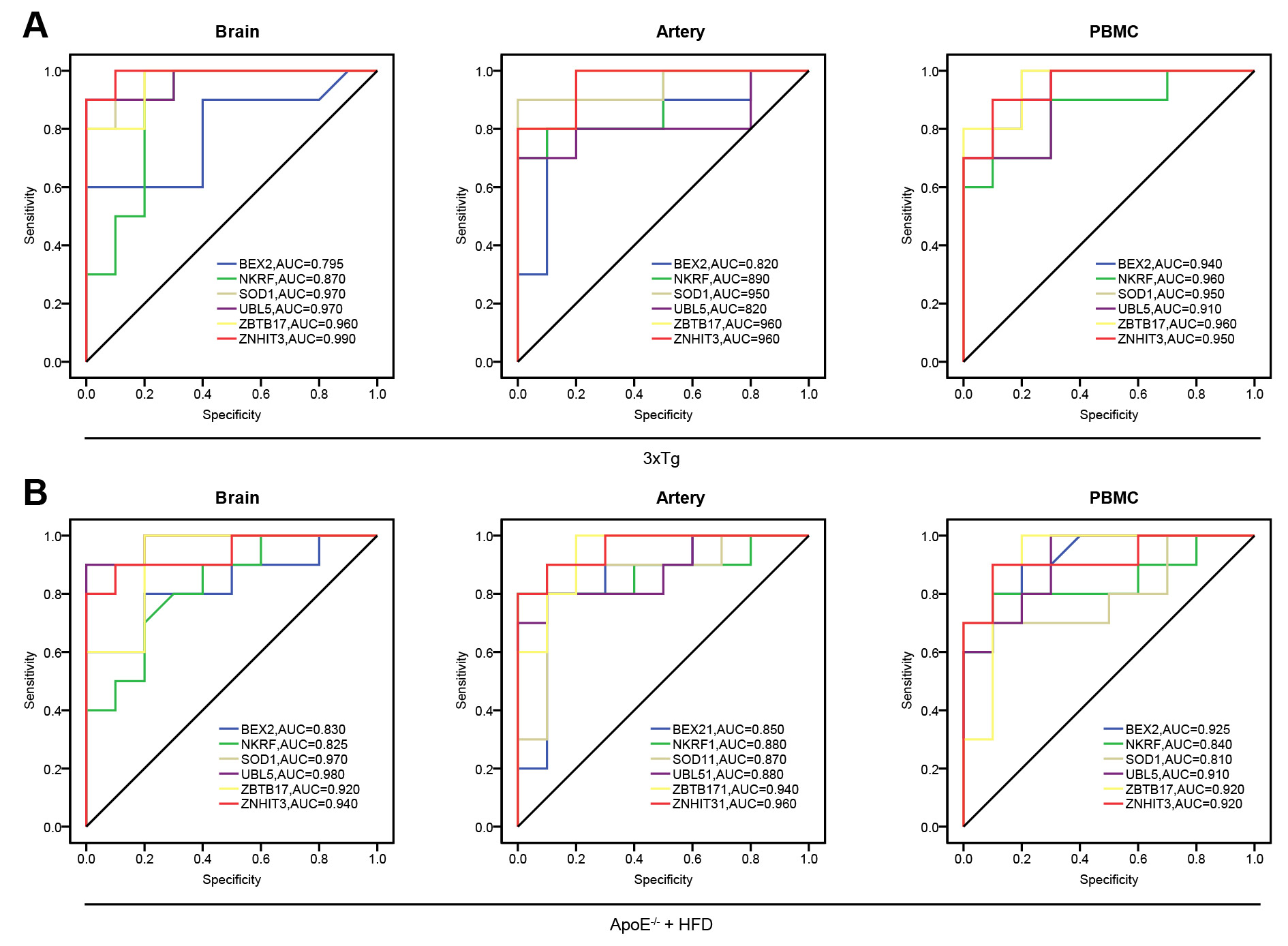

Supplement: Supplementary file 5 — Figure S4. [file CNS-30-e14683-s003.jpg]

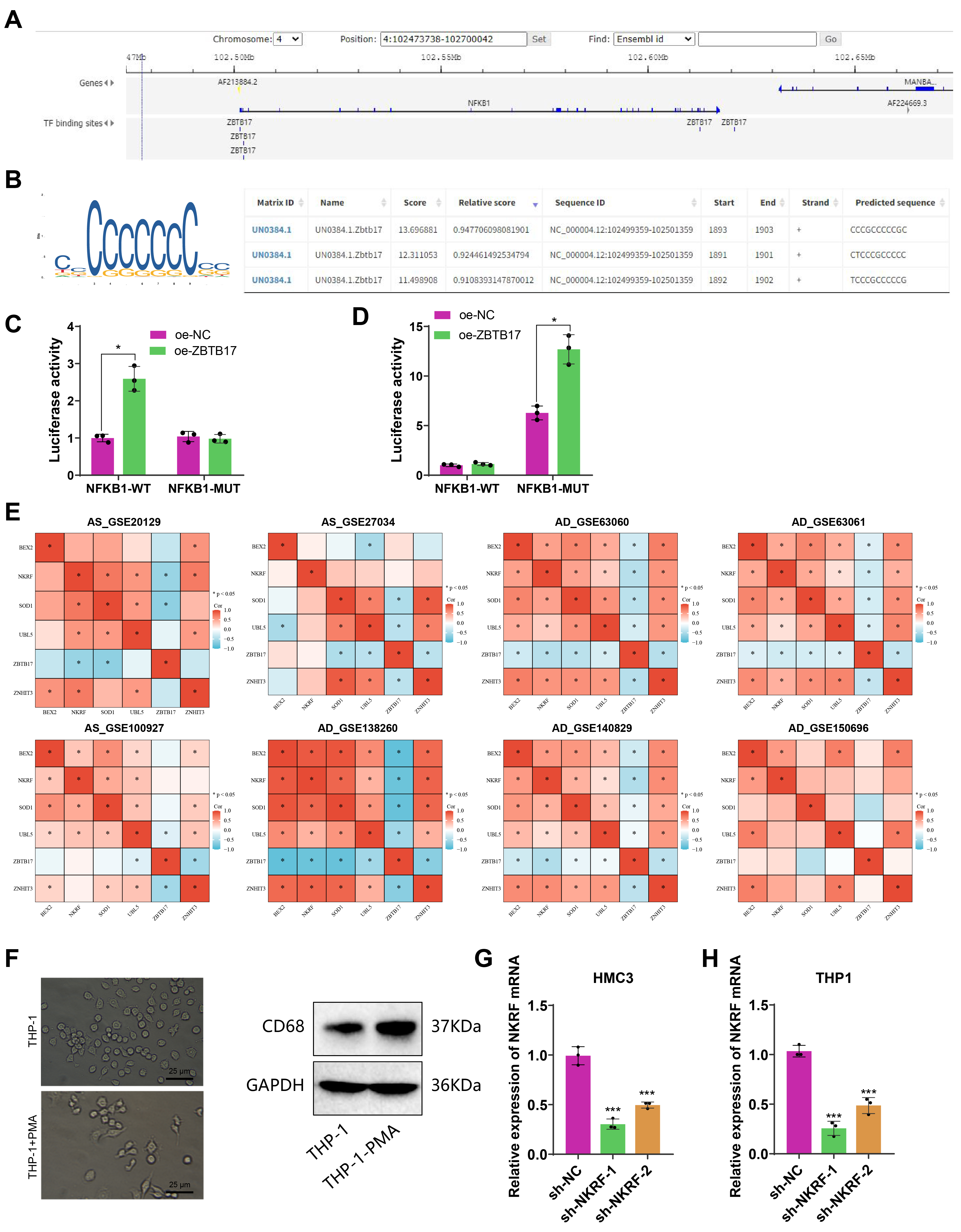

Supplement: Supplementary file 6 — Figure S5. [file CNS-30-e14683-s007.jpg]

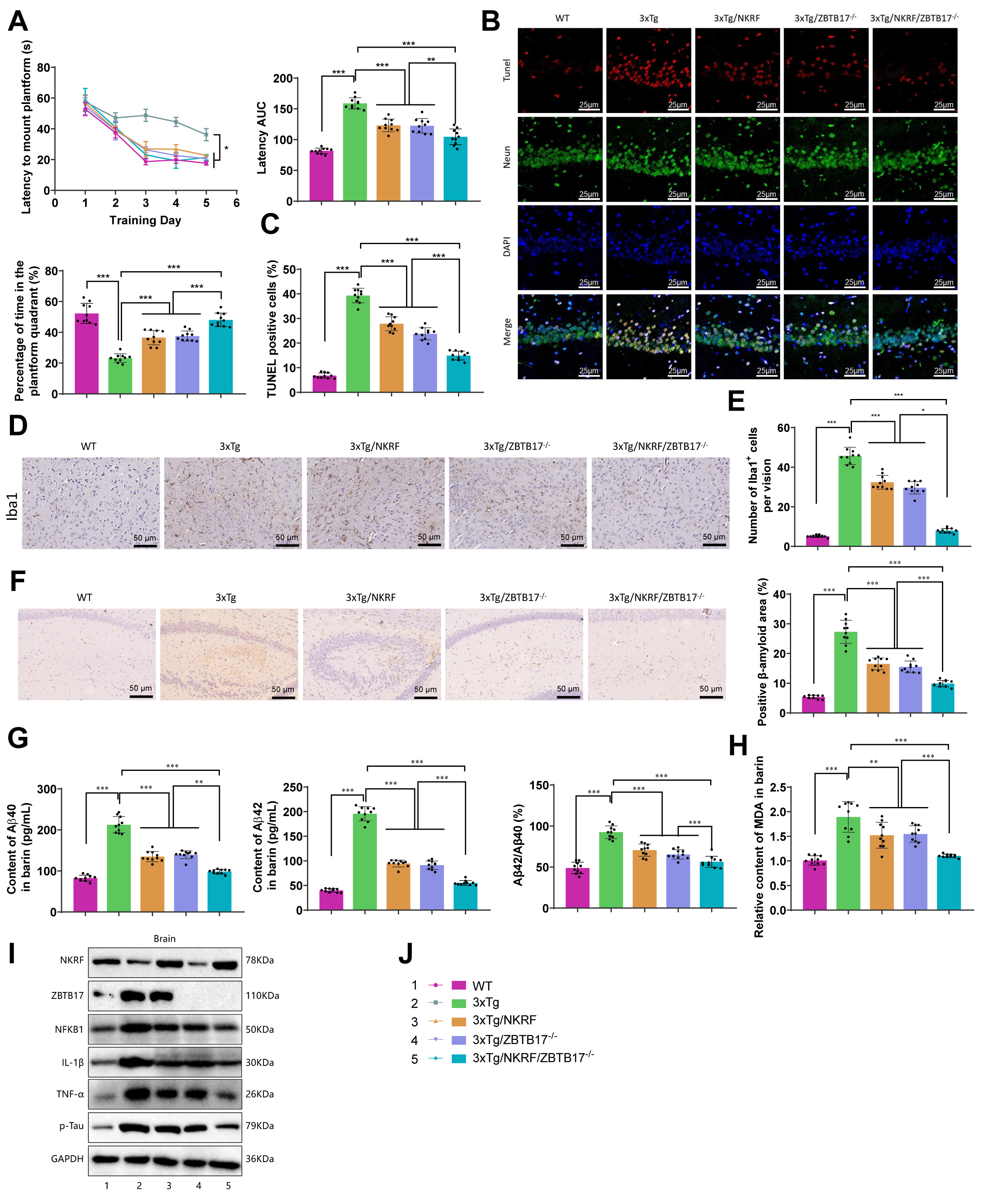

Supplement: Supplementary file 7 — Figure S6. [file CNS-30-e14683-s010.jpg]

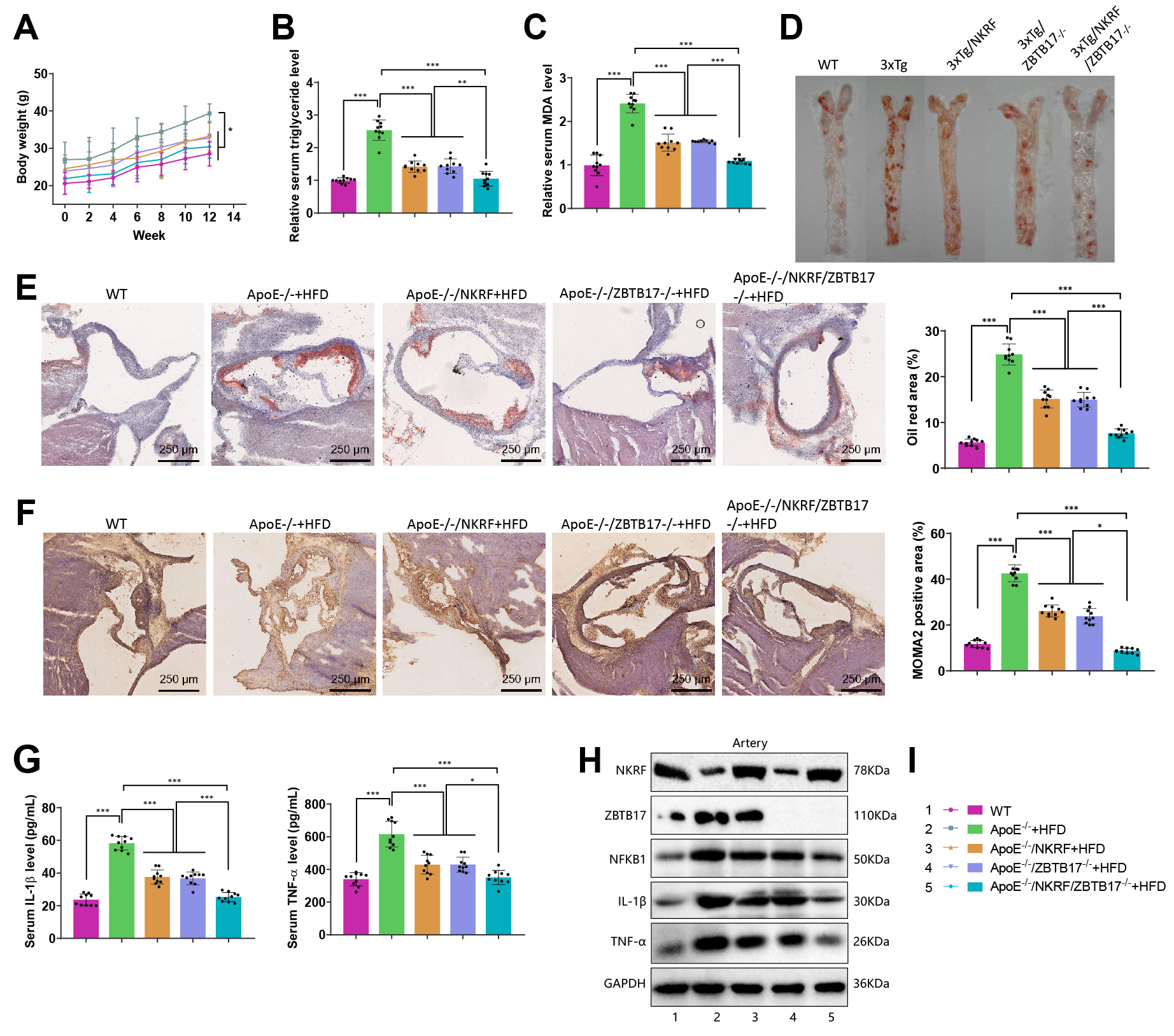

Supplement: Supplementary file 8 — Figure S7. [file CNS-30-e14683-s001.jpg]

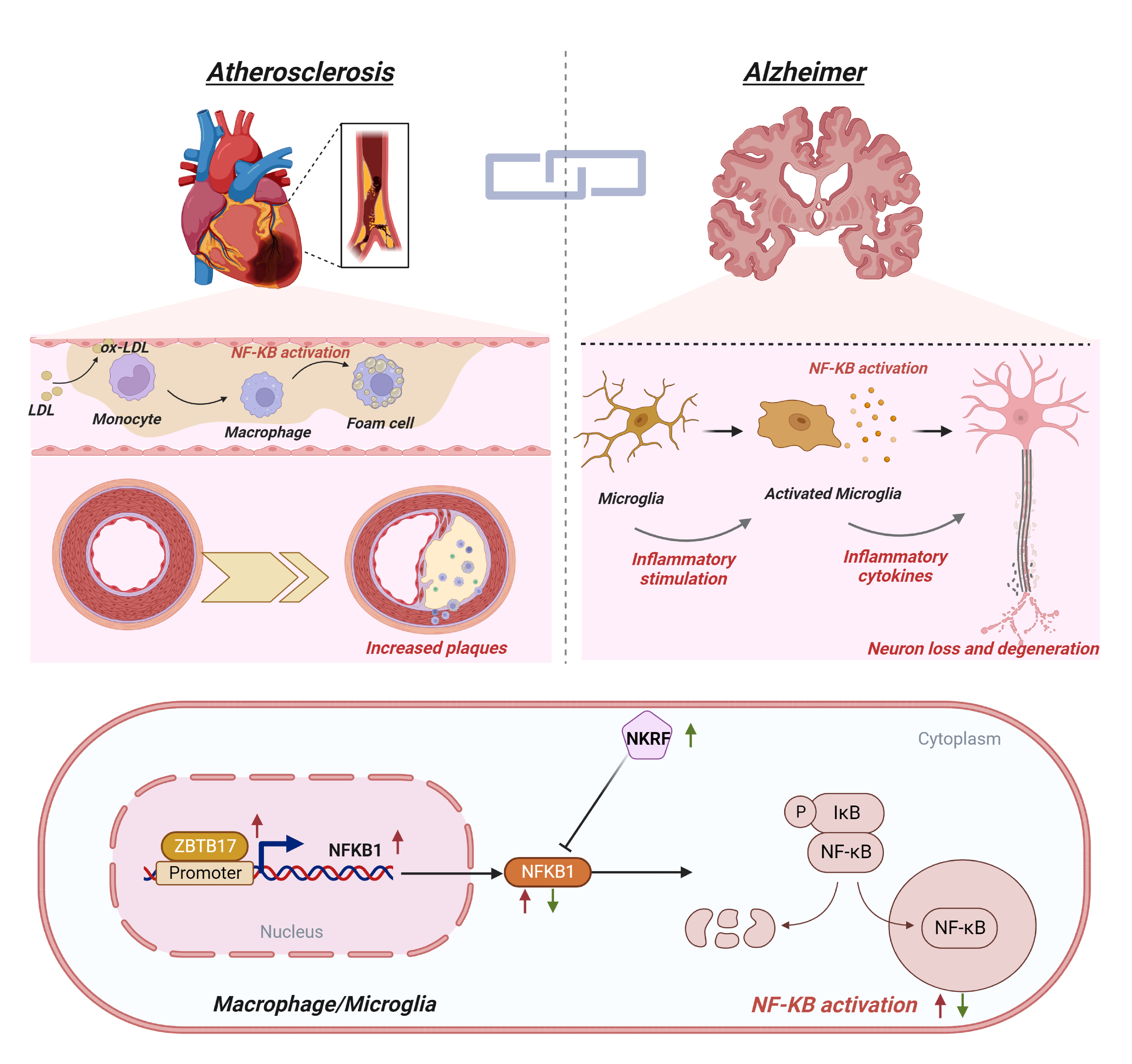

Supplement: Supplementary file 9 — Figure S8. [file CNS-30-e14683-s002.jpg]
