# Supplementary material for: Meta‐analysis and transcriptomic analysis reveal that NKRF and ZBTB17 regulate the NF‐κB signaling pathway, contributing to the shared molecular mechanisms of Alzheimer's disease and atherosclerosis
Source: CNS Neurosci Ther. 2024 May 13;30(5):e14683. doi: 10.1111/cns.14683 (PMC11090078; doi:10.1111/cns.14683)
Supplement: Supplementary file 10 — Table S1. [file CNS-30-e14683-s004.docx]

**Table S1. Geo database chip dataset information**

| **Accession** | **Platform** | **Disease** | **Tissue** | **n (Control)** | **n (Disease)** |
| --- | --- | --- | --- | --- | --- |
| GSE63060 | GPL6947 | AD | PB | 104 | 145 |
| GSE63061 | GPL10558 | AD | PB | 134 | 139 |
| GSE138260 | GPL27556 | AD | Brain | 19 | 17 |
| GSE150696 | GPL17585 | AD | Brain | 9 | 9 |
| GSE140829 | GPL15988 | AD | PB | 249 | 204 |
| GSE20129 | GPL10558 | AS | PB | 8 | 8 |
| GSE100927 | GPL17077 | AS | Artery | 35 | 69 |
| GSE27034 | GPL570 | AS | PBMC | 18 | 19 |

Note: some datasets contain multiple groups of samples, and this study only selected control and ad or as samples for analysis; Pb:peripheral blood; Pbmc:peripheral blood mononuclearcells.

**Table S2. shRNA interference sequence**

| **Name** | **shRNA sequences (5’-3’)** |
| --- | --- |
| sh-NC | CCTAAGGTTAAGTCGCCCTCG |
| sh-NKRF-1 | GCGGAAATTCAAGCATACATT |
| sh-NKRF-2 | GCTTGTGAAGTTAGATGCCAA |

**Table S3. RT-qPCR primer sequences**

| **Genes** | **Sequences (5’-3’)** |
| --- | --- |
| BEX2 (mouse) | F: TCCAAAGTGGAACAAGGCGT |
|  | R: GCACGTAGTAGTCTCCAGCTTC |
| NKRF (mouse) | F: TATTGGGGAGATGCCGTCATA |
|  | R: GGCTTGCTTTTTAGGAGGATTCT |
| NKRF (human) | F: CAGCAGGCAAACTCTCAGTCA |
|  | R: CCAGATTGTCGCCGTTAATTTTT |
| SOD1 (mouse) | F: AACCAGTTGTGTTGTCAGGAC |
|  | R: CCACCATGTTTCTTAGAGTGAGG |
| UBL5 (mouse) | F: AAAGTCCGCGTTAAGTGCAAC |
|  | R: GGTTCATCCCATCGTGGATTTC |
| ZBTB17 (mouse) | F: GACGGCGTTGACTTCAAGG |
|  | R: CGCATTACTGATGTCTAGGTGC |
| ZBTB17 (human) | F: GTGTGATGTGCGGTAAGGC |
|  | R: TGGACTGGACGAATCTCTTGC |
| ZNHIT3 (mouse) | F: ATGGCGTCGCTGAATTGTAGG |
|  | R: AGGGGACCGAACAGTAGGG |
| NFKB1 (human) | F: AACAGAGAGGATTTCGTTTCCG |
|  | R: TTTGACCTGAGGGTAAGACTTCT |
| NFKB2 (human) | F: ATGGAGAGTTGCTACAACCCA |
|  | R: CTGTTCCACGATCACCAGGTA |
| IL-1β (human) | F: ATGATGGCTTATTACAGTGGCAA |
|  | R: GTCGGAGATTCGTAGCTGGA |
| TNF-α (human) | F: CCTCTCTCTAATCAGCCCTCTG |
|  | R: GAGGACCTGGGAGTAGATGAG |
| GAPDH (mouse) | F: AGGTCGGTGTGAACGGATTTG |
|  | R: TGTAGACCATGTAGTTGAGGTCA |
| GAPDH (human) | F: GGAGCGAGATCCCTCCAAAAT |
|  | R: GGCTGTTGTCATACTTCTCATGG |

Note: F, forward; R. Reverse.

**Table S4. Antibody information for Western blot**

| **Name** | **Manufacturer** | **Cat.** | **Dilution ratio** |
| --- | --- | --- | --- |
| BEX2 (mouse) | Thermo Fisher | PA5-100404 | 1:500 |
| NKRF (mouse, human) | Thermo Fisher | PA5-103279 | 1:1000 |
| SOD1 (mouse) | Abcam | ab308181 | 1:1000 |
| UBL5 (mouse) | Abcam | ab192036 | 1:2000 |
| ZBTB17 (mouse, human) | Thermo Fisher | PA5-121200 | 1:500 |
| ZNHIT3(mouse) | Thermo Fisher | PA5-43577 | 1:1000 |
| NFKB1 (human) | Abcam | ab32360 | 1:1000 |
| NFKB2 (human) | Abcam | ab175192 | 1:10000 |
| IL-1β (human) | Abcam | ab254360 | 1:1000 |
| TNF-α (human) | Abcam | ab183218 | 1:1000 |
| p-Tau (mouse) (S396) | Abcam | ab32057 | 1:500 |
| CD68 (mouse) | Abcam | ab283654 | 1:1000 |
| GAPDH (mouse, human) | Abcam | ab8245 | 1:500 |

**Table S5. Meta-analysis results of 18 intersection genes**

| **Gene ID** | **SMD (95%CI)** |
| --- | --- |
| BEX2 * | -0.69 (-0.97; -0.40) |
| C11orf73 * | -0.14 (-0.26; -0.03) |
| CHD8 | 0.12 (-0.15; 0.39) |
| HEATR1 | 0.15 (-0.16; 0.47) |
| HGS | 0.32 (-0.05; 0.68) |
| KLHL17 * | 0.24 (0.12; 0.35) |
| NKRF * | -0.33 (-0.45; -0.22) |
| NLRC5 * | 0.35 (0.23; 0.47) |
| POLR3A | 0.00 (-0.38; 0.38) |
| SEC24C * | 0.36 (0.24; 0.47) |
| SOD1 * | -0.57 (-0.83; -0.32) |
| TTC17 | 0.13 (-0.20; 0.47) |
| TXN | -0.49 (-1.02; 0.03) |
| UBL5 * | -0.53 (-0.81; -0.26) |
| UFC1 * | -0.70 (-1.08; -0.33) |
| VPS16 | 0.25 (-0.05; 0.54) |
| ZBTB17 * | 0.29 (0.17; 0.40) |
| ZNHIT3 * | -0.56 (-0.79; -0.33) |

Note: SMD: standard mean difference; 95%CI: 95% confidence interval; *, means that there is a significant difference in the expression of this gene between groups.
